# Comparative efficacy and safety of first-line treatments for advanced hepatocellular carcinoma: a Bayesian network meta-analysis

**DOI:** 10.3389/fimmu.2026.1846603

**Published:** 2026-06-05

**Authors:** Yun Su, Tongze Cai, Jingxuan Wei, Qiuju Huang, Chun Yao, Lei Fu, Jinghui Zheng, Hongwei Guo, Xiongbin Gui

**Affiliations:** 1 The First Affiliated Hospital, Guangxi University of Chinese Medicine, Nanning, China; 2 Key Laboratory of Longevity and Aging-related Diseases of Chinese Ministry of Education & Guangxi Key Laboratory of Bioactive Molecules Research and Evaluation, Pharmaceutical College, Guangxi Medical University, Nanning, China; 3 The Second Affiliated Hospital, Guangxi University of Chinese Medicine, Nanning, China; 4 Guangxi University of Chinese Medicine, Nanning, China; 5 Guangxi Universities Engineering Research Center for Innovative Product Development of Regional Priority Diseases, Nanning, China

**Keywords:** advanced, Bayesian network meta-analysis, first-line therapy, hepatocellular carcinoma, phase III trials

## Abstract

**Background:**

The therapeutic landscape of advanced hepatocellular carcinoma (HCC) has expanded beyond sorafenib, with multiple combination regimens now demonstrating improved outcomes. However, the absence of direct head-to-head comparisons complicates treatment selection in clinical practice.

**Methods:**

We performed a Bayesian network meta-analysis of phase III randomized controlled trials to compare the efficacy and safety of first-line treatments for patients with advanced HCC. Overall survival (OS), progression-free survival (PFS), and grade ≥ 3 adverse events were assessed. Treatment rankings were estimated using surface under the cumulative ranking curve values.

**Results:**

Sixteen trials comprising 8, 753 patients were included. Hepatic arterial infusion chemotherapy (HAIC)-based regimens, particularly sorafenib plus HAIC and HAIC_FO, achieved the highest rankings for both PFS and OS. Several immunotherapy-based combinations also demonstrated significant survival benefits across multiple clinical subgroups without a corresponding increase in severe adverse events. Subgroup analyses suggested consistent benefits of selected regimens across age, sex, macrovascular invasion, extrahepatic spread, and ECOG performance status.

**Conclusions:**

HAIC-based strategies and selected immunotherapy combinations appear to provide superior survival outcomes as first-line treatment for advanced HCC. Given limitations related to comparator selection and evolving evidence, future head-to-head randomized trials and updated network analyses are warranted to refine optimal treatment strategies.

**Systematic review registration:**

https://www.crd.york.ac.uk/prospero/, identifier CRD420251124082.

## Introduction

Liver cancer remains a major cause of cancer-related death worldwide and ranks third in global cancer mortality (1). In many countries, both the incidence and mortality of liver cancer continue to rise, and its overall prognosis remains poor, with a 5-year survival rate of less than 20% (2). Hepatocellular carcinoma (HCC) represents the most prevalent histological subtype of liver cancer, accounting for nearly 90% of all diagnosed cases. Chronic hepatitis B virus (HBV) infection remains a major etiological driver, contributing to approximately 50% of hepatocellular carcinoma (HCC) cases (3).

For more than a decade, systemic therapy for advanced HCC was largely defined by sorafenib. As the first approved multi-target tyrosine kinase inhibitors (TKIs), sorafenib established a survival benefit but with limited magnitude and durability, in part due to the development of treatment resistance (4). To break through the efficacy bottleneck of sorafenib monotherapy, combination therapy has become an important research direction. Currently, various combination therapies have demonstrated better therapeutic prospects than single-agent treatment in research. Evidence indicates that combining sorafenib with locoregional therapies can further prolong overall survival compared with sorafenib monotherapy (5). In recent years, the treatment paradigm has expanded to include combinations of targeted agents with immune checkpoint inhibitors (6), dual-immune regimens (7), and other novel therapeutic combinations (8).

Clinical studies increasingly support the potential advantages of these approaches. The CLEAP-2302 study indicated that lenvatinib-based triple therapy may confer a survival advantage over bevacizumab-based combinations in unresectable stage IIb–IIIb HCC, although progression-free survival did not differ significantly (9). Similarly, the RATIONALE-208 trial demonstrated sustained antitumor activity and manageable safety with tislelizumab monotherapy in previously treated advanced HCC (10). Despite these advances, response rates to anti–PD-L1–based regimens remain modest, and immune-related toxicities continue to limit their applicability in some patients (11). The immunosuppressive tumor microenvironment characteristic of HCC further compromises antitumor immunity, whereas combining immunotherapy with anti-angiogenic agents may restore immune responsiveness by normalizing tumor vasculature and alleviating hypoxia (12).

As therapeutic options continue to expand, clinicians are increasingly challenged by the absence of direct comparative evidence between first-line regimens. To address this unmet need, we performed a Bayesian network meta-analysis of phase III randomized controlled trials with sorafenib-based control arms to compare the efficacy of first-line treatments for advanced or locally advanced HCC and to support evidence-based treatment selection.

## Methods

This systematic review and Bayesian network meta-analysis was performed in line with PRISMA-NMA (13) recommendations and registered in PROSPERO (CRD420251124082).

### Search strategy

A comprehensive literature search of PubMed, EMBASE, Web of Science, Cochrane CENTRAL, and ClinicalTrials.gov was conducted up to August 11, 2025. We included English-language phase III randomized controlled trials. Abstract-only records were excluded if full reports were subsequently available. Full search details are provided in **Supplementary Table 1**.

### Eligibility criteria

We included trials enrolling patients with advanced or unresectable hepatocellular carcinoma that used sorafenib as the comparator. Eligible interventions encompassed systemic treatments, locoregional therapies, and combination regimens. Studies were required to report survival outcomes, such as progression-free survival (PFS), overall survival (OS), as well as grade ≥ 3 adverse events.

Trials lacking relevant outcomes, using non-randomized designs, or comparing regimens within the same treatment class were excluded.

### Data extraction and bias assessment

Two reviewers (Su and Cai) independently extracted study characteristics, patient baseline features, treatment regimens, and outcome data. Discrepancies were resolved through discussion with a third reviewer (Wei). The risk of bias for all included studies was systematically assessed using the revised Cochrane Risk-of-Bias Tool (RoB 2) (14).

### Statistical analysis

A Bayesian network meta-analysis was performed using R (version 4.4.1) with the gemtc package (15) and JAGS (rjags package (16)). Hazard ratios (HR) with 95% Confidence interval (CI) were applied for OS and PFS, and odds ratios (OR) for grade ≥ 3 adverse events. Model estimation was based on Markov chain Monte Carlo (MCMC) methods (17) with convergence assessed by standard diagnostics. Three independent chains were run, with 20, 000 burn-in iterations for model adaptation followed by 50, 000 sampling iterations for stable posterior estimates. Convergence across chains was assessed using trace plots, density plots, and the Gelman–Rubin diagnostic. Treatment rankings were summarized using SUCRA values (18). Between-study heterogeneity was evaluated using the I² statistic (19). I² < 25% was considered low heterogeneity, 25%–50% moderate, and > 50% substantial heterogeneity. A random-effects model was applied when heterogeneity was evident; otherwise, a fixed-effect model was used when I² ≤ 50%.

## Results

### Study selection and characteristics

**Figure 1:**
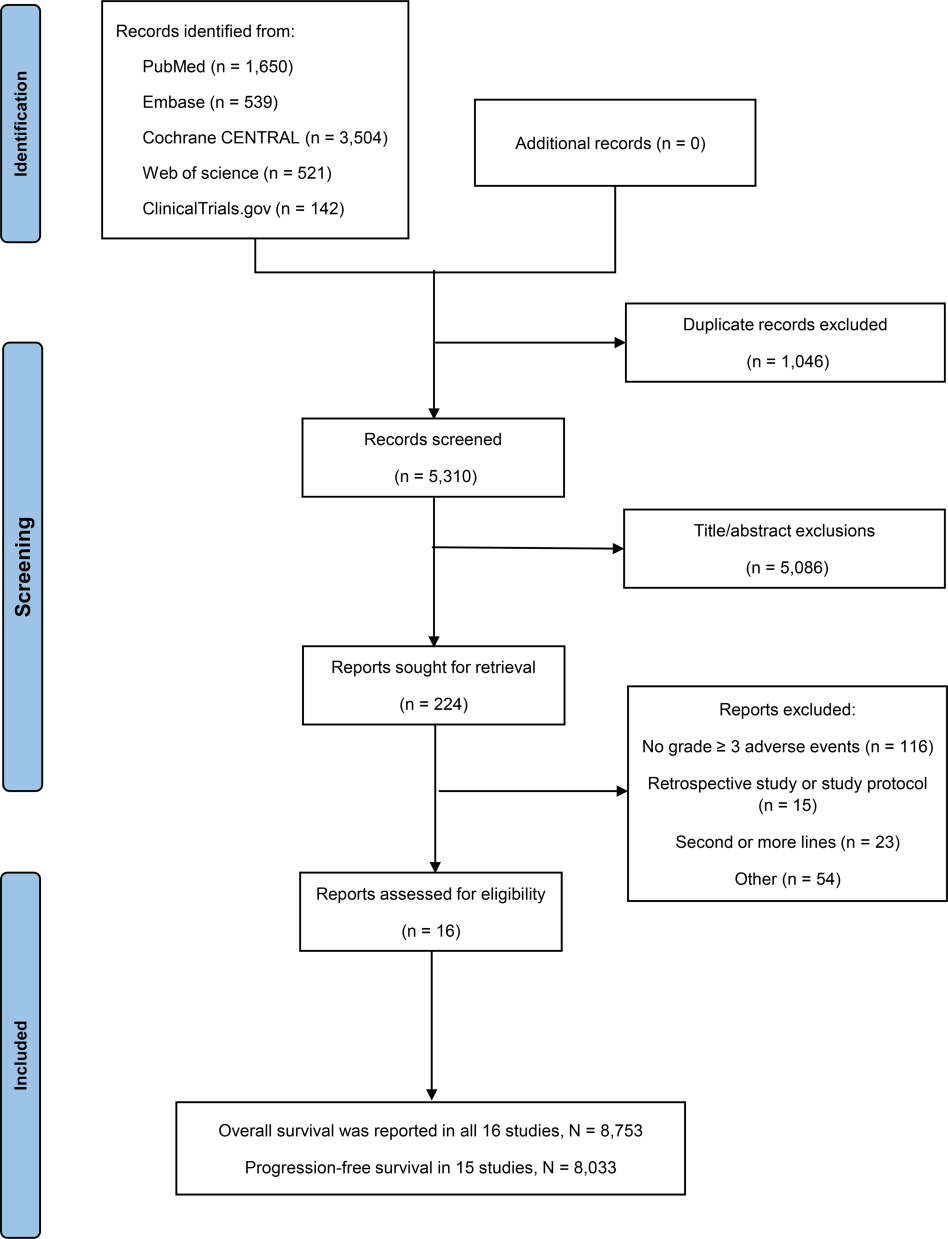
Overview of the research process.

A total of 6, 356 records were identified through database searches searching, of which 16 (20–35) phase III randomized controlled trials met the eligibility criteria (**Figure 1**). These included two three-arm trials and thirteen two-arm trials. Overall survival data were available for all 8, 753 enrolled patients, while 15 studies additionally reported progression-free survival outcomes for 8, 033 patients.

**Figure 2:**
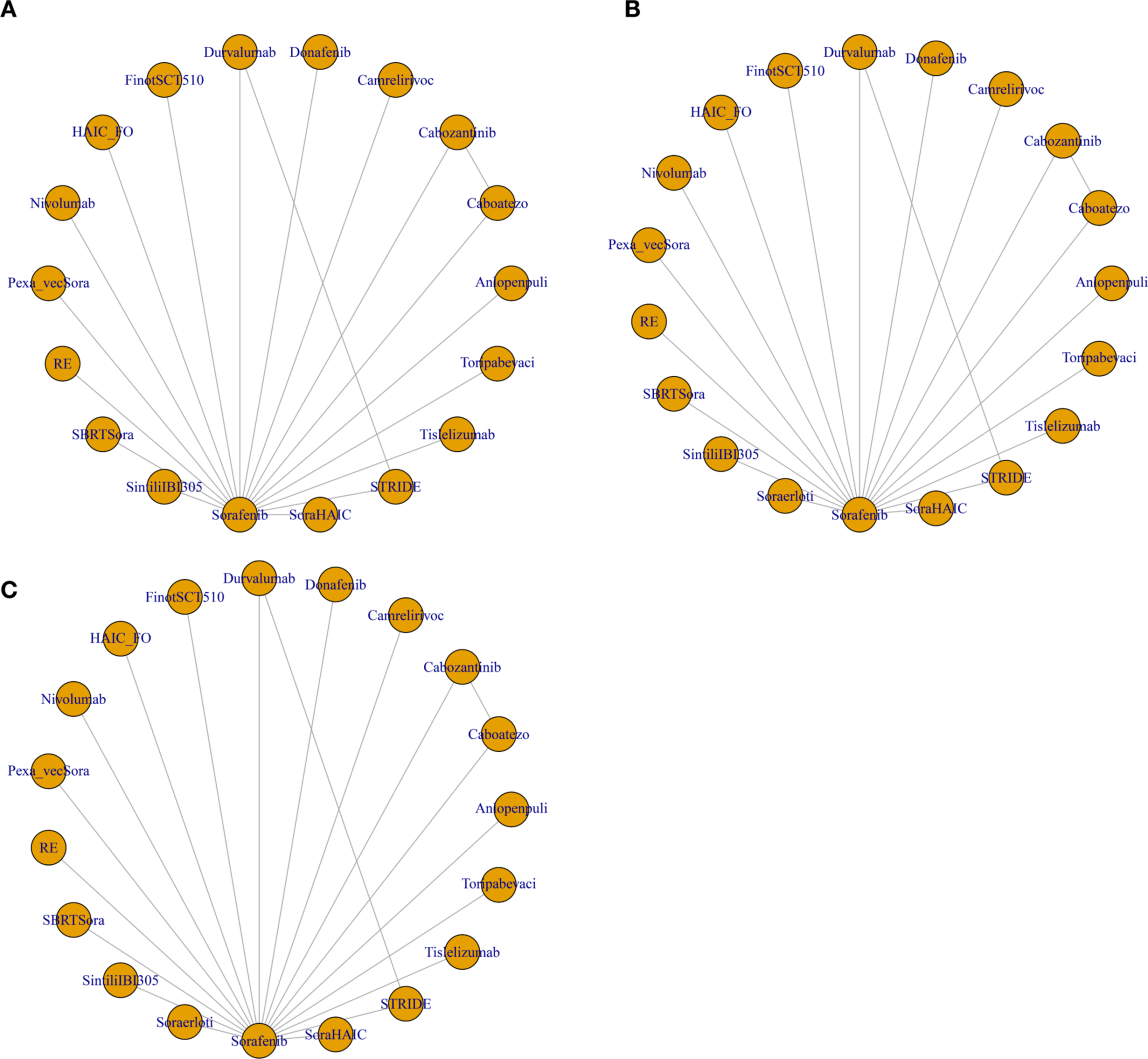
Network diagrams depicting direct and indirect comparisons among first-line therapies for advanced or unresectable hepatocellular carcinoma. **(A)** progression-free survival, **(B)** overall survival, and **(C)** grade ≥ 3 adverse events. Soraerloti, Sorafenib + erlotinib; SoraHAIC, Sorafenib + hepatic arterial infusion chemotherapy; SintiliIBI305, Sintilimab + Bevacizumab biosimilar; STRIDE, Single Tremelimumab Regular Interval Durvalumab; HAIC_FO, Hepatic arterial infusion chemotherapy of infusional fluorouracil; Camrelirivoc, Camrelizumab + rivoceranib; Pexa_vecSora, Pexastimogene devacirepvec + sorafenib; Caboatezo, Cabozantinib + atezolizumab; SBRTSora, Stereotactic body radiation therapy + sorafenib; FinotSCT510, Finotonlimab + bevacizumab biosimilar; Toripabevaci, Toripalimab + bevacizumab; Anlopenpuli, Anlotinib + penpulimab; RE, radioembolization.

This study systematically compared first-line treatment strategies for advanced or unresectable locally advanced hepatocellular carcinoma, encompassing systemic therapy alone, locoregional approaches, and combinations of locoregional and systemic treatments. Beyond molecular targeted therapies represented by sorafenib, the analysis also included immunotherapy- and anti-angiogenic–based regimens, as well as their combinations, to comprehensively evaluate differences in therapeutic efficacy across treatment modalities. Network structures for PFS, OS, and grade ≥ 3 adverse events are presented in **Figures 2A–C**. Risk-of-bias assessment using the RoB 2 tool is summarized in **Supplementary Figures 1A, B**.

### Efficacy outcomes

Pairwise comparisons were conducted for progression-free survival, overall survival, and grade ≥3 adverse events. Adequate model convergence was confirmed for all outcomes based on trace plots and Brooks–Gelman–Rubin diagnostics (**Supplementary Figures 2**, **3**).Regarding progression-free survival, most combination or novel regimens demonstrated superior efficacy compared with sorafenib monotherapy. Significant reductions in the risk of disease progression were observed with SoraHAIC (HR = 0.33, 95% CI: 0.25–0.43), HAIC_FO (HR = 0.45, 95% CI: 0.34–0.60), FinotSCT510 (HR = 0.50, 95% CI: 0.38–0.65), Anlopenpuli (HR = 0.52, 95% CI: 0.41–0.66), Camrelirivoc (HR = 0.52, 95% CI: 0.41–0.65), SBRTSora (HR = 0.55, 95% CI: 0.40–0.75), SintiliIBI305 (HR = 0.56, 95% CI: 0.45–0.69), Toripabevaci (HR = 0.69, 95% CI: 0.53–0.90), and Caboatezo (HR = 0.74, 95% CI: 0.56–0.97).

In contrast, Cabozantinib (HR = 0.78, 95% CI: 0.56–1.09), STRIDE (HR = 0.90, 95% CI: 0.77–1.05), RE (HR = 0.89, 95% CI: 0.71–1.12), and Donafenib (HR = 0.91, 95% CI: 0.76–1.08) did not show meaningful PFS improvement over sorafenib. Similarly, no significant differences in PFS were observed when sorafenib was compared with Durvalumab (HR = 0.98, 95% CI: 0.84–1.14), Nivolumab (HR = 0.97, 95% CI: 0.79–1.20), Tislelizumab (HR = 0.90, 95% CI: 0.75–1.08), or Pexa_vecSora (HR = 0.82, 95% CI: 0.65–1.04) (**Table 1**).

**Table 1:** League table of progression-free survival for first-line treatments in advanced or unresectable hepatocellular carcinoma.

Progression-free survival																	
*Rank1*																	
**SoraHAIC**	*Rank2*																
0.73 (0.49, 1.08)	**HAIC_FO**	*Rank3*															
0.66 (0.45, 0.97)	0.90 (0.61, 1.33)	**FinotSCT510**	*Rank4*														
0.63 (0.44, 0.91)	0.87 (0.60, 1.25)	0.96 (0.67, 1.37)	**Camrelirivoc**	*Rank5*													
0.63 (0.44, 0.91)	0.87 (0.60, 1.25)	0.96 (0.67, 1.38)	1.00 (0.72, 1.39)	**Anlopenpuli**	*Rank6*												
0.60 (0.40, 0.91)	0.82 (0.54, 1.25)	0.91 (0.60, 1.38)	0.94 (0.64, 1.39)	0.94 (0.64, 1.40)	**SBRTSora**	*Rank7*											
0.59 (0.42, 0.83)	0.81 (0.57, 1.15)	0.89 (0.63, 1.26)	0.93 (0.68, 1.27)	0.93 (0.68, 1.28)	0.98 (0.67, 1.43)	**SintiliIBI305**	*Rank8*										
0.48 (0.33, 0.70)	0.65 (0.44, 0.97)	0.72 (0.49, 1.06)	0.75 (0.53, 1.07)	0.75 (0.53, 1.08)	0.80 (0.53, 1.20)	0.81 (0.58, 1.14)	**Toripabevaci**	*Rank9*									
0.45 (0.30, 0.66)	0.61 (0.41, 0.90)	0.68 (0.46, 0.99)	0.70 (0.49, 1.01)	0.70 (0.49, 1.01)	0.74 (0.49, 1.13)	0.76 (0.54, 1.07)	0.93 (0.64, 1.37)	**Caboatezo**	*Rank10*								
0.42 (0.28, 0.65)	0.58 (0.37, 0.90)	0.64 (0.42, 0.99)	0.67 (0.45, 1.00)	0.67 (0.44, 1.00)	0.71 (0.45, 1.12)	0.72 (0.49, 1.07)	0.89 (0.58, 1.36)	0.95 (0.72, 1.24)	**Cabozantinib**	*Rank11*							
0.37 (0.27, 0.50)	0.50 (0.36, 0.69)	0.56 (0.41, 0.76)	0.58 (0.44, 0.76)	0.58 (0.43, 0.77)	0.61 (0.43, 0.87)	0.62 (0.48, 0.81)	0.77 (0.56, 1.05)	0.82 (0.60, 1.13)	0.87 (0.60, 1.25)	**STRIDE**	*Rank12*						
0.37 (0.26, 0.53)	0.51 (0.35, 0.73)	0.56 (0.40, 0.80)	0.58 (0.42, 0.81)	0.58 (0.42, 0.81)	0.62 (0.42, 0.91)	0.63 (0.46, 0.86)	0.78 (0.55, 1.10)	0.83 (0.58, 1.18)	0.88 (0.59, 1.31)	1.01 (0.77, 1.33)	**RE**	*Rank13*					
0.36 (0.26, 0.50)	0.50 (0.36, 0.69)	0.55 (0.40, 0.76)	0.57 (0.43, 0.77)	0.57 (0.43, 0.77)	0.61 (0.42, 0.87)	0.62 (0.47, 0.81)	0.76 (0.55, 1.05)	0.81 (0.59, 1.13)	0.86 (0.59, 1.25)	0.99 (0.78, 1.25)	0.98 (0.74, 1.30)	**Donafenib**	*Rank14*				
0.33 (0.25, 0.43)	0.45 (0.34, 0.60)	0.50 (0.38, 0.65)	0.52 (0.41, 0.65)	0.52 (0.41, 0.66)	0.55 (0.40, 0.75)	0.56 (0.45, 0.69)	0.69 (0.53, 0.90)	0.74 (0.56, 0.97)	0.78 (0.56, 1.09)	0.90 (0.77, 1.05)	0.89 (0.71, 1.12)	0.91 (0.76, 1.08)	**Sorafenib**	*Rank15*			
0.32 (0.24, 0.44)	0.44 (0.32, 0.61)	0.49 (0.36, 0.67)	0.51 (0.39, 0.67)	0.51 (0.38, 0.68)	0.54 (0.38, 0.76)	0.55 (0.42, 0.71)	0.68 (0.50, 0.92)	0.73 (0.53, 0.99)	0.76 (0.53, 1.10)	0.88 (0.76, 1.03)	0.87 (0.67, 1.15)	0.89 (0.71, 1.12)	0.98 (0.84, 1.14)	**Durvalumab**	*Rank16*		
0.32 (0.23, 0.45)	0.44 (0.31, 0.62)	0.49 (0.35, 0.68)	0.50 (0.37, 0.69)	0.50 (0.37, 0.70)	0.53 (0.37, 0.78)	0.54 (0.40, 0.73)	0.67 (0.48, 0.94)	0.72 (0.51, 1.02)	0.76 (0.51, 1.12)	0.87 (0.67, 1.14)	0.86 (0.63, 1.18)	0.88 (0.67, 1.16)	0.97 (0.79, 1.20)	0.99 (0.77, 1.28)	**Nivolumab**	*Rank17*	
0.30 (0.21, 0.41)	0.41 (0.29, 0.57)	0.45 (0.33, 0.62)	0.47 (0.35, 0.63)	0.47 (0.35, 0.63)	0.50 (0.34, 0.71)	0.50 (0.38, 0.67)	0.62 (0.45, 0.86)	0.67 (0.48, 0.93)	0.70 (0.48, 1.03)	0.81 (0.64, 1.03)	0.80 (0.60, 1.07)	0.82 (0.64, 1.06)	0.90 (0.75, 1.08)	0.92 (0.72, 1.17)	0.93 (0.70, 1.23)	**Tislelizumab**	*Rank18*
0.27 (0.19, 0.39)	0.37 (0.26, 0.53)	0.41 (0.29, 0.59)	0.43 (0.31, 0.59)	0.43 (0.31, 0.60)	0.45 (0.31, 0.67)	0.46 (0.34, 0.63)	0.57 (0.40, 0.81)	0.61 (0.42, 0.87)	0.64 (0.43, 0.96)	0.74 (0.56, 0.98)	0.73 (0.53, 1.01)	0.75 (0.56, 1.00)	0.82 (0.65, 1.04)	0.84 (0.64, 1.10)	0.85 (0.62, 1.16)	0.91 (0.68, 1.23)	**Pexa_vecSora**

HR below 1.00 reflects a survival benefit. Soraerloti, Sorafenib + erlotinib; SoraHAIC, Sorafenib + hepatic arterial infusion chemotherapy; SintiliIBI305, Sintilimab + Bevacizumab biosimilar; STRIDE, Single Tremelimumab Regular Interval Durvalumab; HAIC_FO, Hepatic arterial infusion chemotherapy of infusional fluorouracil; Camrelirivoc, Camrelizumab + rivoceranib; Pexa_vecSora, Pexastimogene devacirepvec + sorafenib; Caboatezo, Cabozantinib + atezolizumab; SBRTSora, Stereotactic body radiation therapy + sorafenib; FinotSCT510, Finotonlimab + bevacizumab biosimilar; Toripabevaci, Toripalimab + bevacizumab; Anlopenpuli, Anlotinib + penpulimab. RE, radioembolization.

Bold font indicates treatment names.

Regarding overall survival, several regimens demonstrated significant survival advantages over sorafenib monotherapy. Notably, SoraHAIC (HR = 0.35, 95% CI: 0.26–0.48), HAIC_FO (HR = 0.41, 95% CI: 0.30–0.55), SintiliIBI305 (HR = 0.57, 95% CI: 0.43–0.75), FinotSCT510 (HR = 0.60, 95% CI: 0.44–0.81), Camrelirivoc (HR = 0.62, 95% CI: 0.49–0.79), Anlopenpuli (HR = 0.69, 95% CI: 0.55–0.87), Toripabevaci (HR = 0.76, 95% CI: 0.58–0.99), STRIDE (HR = 0.78, 95% CI: 0.65–0.93), and Donafenib (HR = 0.83, 95% CI: 0.70–0.99) all yielded a statistically significant reduction in mortality risk.

In contrast, SBRTSora (HR = 0.77, 95% CI: 0.59–1.01), Nivolumab (HR = 0.85, 95% CI: 0.71–1.01), Tislelizumab (HR = 0.85, 95% CI: 0.71–1.02), Durvalumab (HR = 0.86, 95% CI: 0.72–1.02), Cabozantinib (HR = 0.90, 95% CI: 0.69–1.18), Soraerloti (HR = 0.93, 95% CI: 0.78–1.11), and Caboatezo (HR = 0.98, 95% CI: 0.78–1.24) did not demonstrate meaningful OS benefits relative to sorafenib. Similarly, no significant survival difference was observed in the comparison between sorafenib and RE (HR = 0.89, 95% CI: 0.72–1.11), sorafenib and Pexa_vecSora (HR = 0.84, 95% CI: 0.65–1.08) (**Table 2**).

**Table 2:** League table of overall survival for first-line treatments in advanced or unresectable hepatocellular carcinoma.

Overall survival																		
*Rank1*																		
**SoraHAIC**	*Rank2*																	
0.86 (0.56, 1.32)	**HAIC_FO**	*Rank3*																
0.61 (0.41, 0.93)	0.72 (0.47, 1.08)	**SintiliIBI305**	*Rank4*															
0.58 (0.38, 0.90)	0.68 (0.44, 1.05)	0.95 (0.63, 1.43)	**FinotSCT510**	*Rank5*														
0.56 (0.38, 0.84)	0.66 (0.44, 0.97)	0.92 (0.63, 1.33)	0.97 (0.66, 1.43)	**Camrelirivoc**	*Rank6*													
0.51 (0.35, 0.74)	0.59 (0.40, 0.87)	0.83 (0.58, 1.19)	0.87 (0.59, 1.27)	0.90 (0.64, 1.26)	**Anlopenpuli**	*Rank7*												
0.46 (0.31, 0.69)	0.54 (0.36, 0.81)	0.75 (0.51, 1.10)	0.79 (0.53, 1.19)	0.82 (0.57, 1.17)	0.91 (0.64, 1.29)	**Toripabevaci**	*Rank8*											
0.45 (0.32, 0.64)	0.52 (0.37, 0.75)	0.73 (0.53, 1.02)	0.77 (0.54, 1.10)	0.80 (0.59, 1.08)	0.89 (0.66, 1.19)	0.98 (0.71, 1.35)	**STRIDE**	*Rank9*										
0.45 (0.30, 0.68)	0.53 (0.35, 0.79)	0.74 (0.50, 1.09)	0.78 (0.52, 1.17)	0.80 (0.56, 1.16)	0.90 (0.63, 1.28)	0.99 (0.68, 1.44)	1.01 (0.73, 1.4)	**SBRTSora**	*Rank10*									
0.42 (0.30, 0.60)	0.49 (0.35, 0.70)	0.69 (0.49, 0.95)	0.72 (0.51, 1.03)	0.75 (0.55, 1.01)	0.83 (0.62, 1.11)	0.91 (0.67, 1.26)	0.94 (0.73, 1.2)	0.93 (0.67, 1.28)	**Donafenib**	*Rank11*								
0.41 (0.29, 0.59)	0.48 (0.34, 0.68)	0.67 (0.48, 0.93)	0.71 (0.50, 1.00)	0.73 (0.54, 0.99)	0.81 (0.61, 1.08)	0.89 (0.65, 1.23)	0.92 (0.72, 1.18)	0.91 (0.66, 1.25)	0.98 (0.77, 1.25)	**Nivolumab**	*Rank12*							
0.41 (0.29, 0.59)	0.48 (0.34, 0.68)	0.67 (0.48, 0.93)	0.71 (0.49, 1.01)	0.73 (0.54, 0.99)	0.81 (0.61, 1.09)	0.89 (0.65, 1.24)	0.92 (0.71, 1.18)	0.91 (0.65, 1.25)	0.98 (0.76, 1.26)	1.00 (0.78, 1.29)	**Tislelizumab**	*Rank13*						
0.41 (0.29, 0.58)	0.47 (0.33, 0.67)	0.66 (0.48, 0.92)	0.70 (0.49, 0.99)	0.72 (0.53, 0.97)	0.80 (0.60, 1.07)	0.88 (0.64, 1.22)	0.91 (0.76, 1.08)	0.90 (0.65, 1.23)	0.97 (0.76, 1.23)	0.99 (0.77, 1.26)	0.99 (0.77, 1.27)	**Durvalumab**	*Rank14*					
0.39 (0.26, 0.58)	0.45 (0.30, 0.68)	0.63 (0.43, 0.93)	0.67 (0.44, 1.00)	0.69 (0.48, 0.99)	0.77 (0.54, 1.09)	0.84 (0.58, 1.23)	0.87 (0.63, 1.20)	0.86 (0.58, 1.25)	0.92 (0.67, 1.27)	0.95 (0.69, 1.30)	0.94 (0.68, 1.31)	0.96 (0.69, 1.31)	**Cabozantinib**	*Rank15*				
0.38 (0.26, 0.54)	0.44 (0.31, 0.62)	0.61 (0.44, 0.85)	0.65 (0.45, 0.92)	0.67 (0.49, 0.90)	0.74 (0.56, 0.99)	0.82 (0.60, 1.12)	0.84 (0.65, 1.08)	0.83 (0.60, 1.14)	0.89 (0.70, 1.14)	0.91 (0.71, 1.17)	0.91 (0.71, 1.17)	0.92 (0.72, 1.18)	0.97 (0.70, 1.33)	**Soraerloti**	*Rank16*			
0.36 (0.24, 0.52)	0.42 (0.28, 0.61)	0.58 (0.41, 0.84)	0.61 (0.42, 0.90)	0.63 (0.45, 0.89)	0.70 (0.51, 0.98)	0.78 (0.54, 1.10)	0.80 (0.59, 1.07)	0.79 (0.55, 1.12)	0.85 (0.63, 1.13)	0.87 (0.65, 1.16)	0.87 (0.65, 1.16)	0.88 (0.66, 1.17)	0.92 (0.74, 1.15)	0.95 (0.71, 1.27)	**Caboatezo**	*Rank17*		
0.35 (0.26, 0.48)	0.41 (0.30, 0.55)	0.57 (0.43, 0.75)	0.60 (0.44, 0.81)	0.62 (0.49, 0.79)	0.69 (0.55, 0.87)	0.76 (0.58, 0.99)	0.78 (0.65, 0.93)	0.77 (0.59, 1.01)	0.83 (0.70, 0.99)	0.85 (0.71, 1.01)	0.85 (0.71, 1.02)	0.86 (0.72, 1.02)	0.90 (0.69, 1.18)	0.93 (0.78, 1.11)	0.98 (0.78, 1.24)	**Sorafenib**	*Rank18*	
0.31 (0.21, 0.46)	0.36 (0.25, 0.53)	0.51 (0.36, 0.73)	0.54 (0.37, 0.78)	0.55 (0.40, 0.77)	0.62 (0.45, 0.85)	0.68 (0.48, 0.96)	0.70 (0.52, 0.92)	0.69 (0.49, 0.97)	0.74 (0.56, 0.98)	0.76 (0.57, 1.01)	0.76 (0.57, 1.01)	0.77 (0.58, 1.02)	0.80 (0.57, 1.14)	0.83 (0.63, 1.10)	0.87 (0.63, 1.20)	0.89 (0.72, 1.11)	**RE**	*Rank19*
0.29 (0.20, 0.44)	0.34 (0.23, 0.51)	0.48 (0.33, 0.69)	0.50 (0.34, 0.75)	0.52 (0.37, 0.74)	0.58 (0.41, 0.81)	0.64 (0.44, 0.92)	0.65 (0.48, 0.89)	0.65 (0.45, 0.93)	0.70 (0.51, 0.95)	0.71 (0.52, 0.97)	0.71 (0.52, 0.97)	0.72 (0.53, 0.98)	0.75 (0.52, 1.09)	0.78 (0.57, 1.06)	0.82 (0.58, 1.15)	0.84 (0.65, 1.08)	0.94 (0.67, 1.31)	**Pexa_vecSora**

HR below 1.00 reflects a survival benefit. Soraerloti, Sorafenib + erlotinib; SoraHAIC, Sorafenib + hepatic arterial infusion chemotherapy; SintiliIBI305, Sintilimab + Bevacizumab biosimilar; STRIDE, Single Tremelimumab Regular Interval Durvalumab; HAIC_FO, Hepatic arterial infusion chemotherapy of infusional fluorouracil; Camrelirivoc, Camrelizumab + rivoceranib; Pexa_vecSora, Pexastimogene devacirepvec + sorafenib; Caboatezo, Cabozantinib + atezolizumab; SBRTSora, Stereotactic body radiation therapy + sorafenib; FinotSCT510, Finotonlimab + bevacizumab biosimilar; Toripabevaci, Toripalimab + bevacizumab; Anlopenpuli, Anlotinib + penpulimab. RE, radioembolization.

Bold font indicates treatment names.

Notably, when Camrelirivoc was used as the reference, Tislelizumab (OR = 0.06, 95% CI: 0.01–0.78), HAIC_FO (OR = 0.07, 95% CI: 0.01–0.87), and Nivolumab (OR = 0.08, 95% CI: 0.01–0.94) were associated with significantly lower odds of grade ≥ 3 treatment-related adverse events. Apart from these comparisons, no statistically significant differences in the risk of grade ≥ 3 adverse events were observed among the remaining treatment regimens, including comparisons with sorafenib (**Table 3**). These findings indicate that, while several treatments offer superior clinical efficacy, they do so without increasing the risk of severe adverse events, suggesting an overall acceptable safety profile.

**Table 3:** League table of 3 and above adverse events for first-line treatments in advanced or unresectable hepatocellular carcinoma.

Grade ≥ 3 adverse events																		
*Rank1*																		
**Tislelizumab**	*Rank2*																	
0.92 (0.07, 11.00)	**HAIC_FO**	*Rank3*																
0.82 (0.07, 10.00)	0.9 (0.07, 11.52)	**Nivolumab**	*Rank4*															
0.66 (0.05, 7.91)	0.72 (0.06, 9.23)	0.81 (0.07, 9.98)	**RE**	*Rank5*														
0.46 (0.04, 5.30)	0.50 (0.04, 6.11)	0.56 (0.05, 6.57)	0.69 (0.06, 8.25)	**Durvalumab**	*Rank6*													
0.41 (0.03, 4.84)	0.44 (0.04, 5.63)	0.49 (0.04, 5.94)	0.61 (0.05, 7.53)	0.88 (0.07, 10.43)	**Donafenib**	*Rank7*												
0.32 (0.03, 3.90)	0.34 (0.03, 4.58)	0.39 (0.03, 4.78)	0.48 (0.04, 5.83)	0.69 (0.06, 8.42)	0.78 (0.06, 9.42)	**Soraerloti**	*Rank8*											
0.27 (0.02, 3.08)	0.29 (0.02, 3.59)	0.32 (0.03, 3.79)	0.40 (0.03, 4.75)	0.58 (0.10, 3.30)	0.65 (0.05, 7.54)	0.84 (0.07, 10.03)	**STRIDE**	*Rank9*										
0.25 (0.04, 1.38)	0.27 (0.05, 1.64)	0.30 (0.05, 1.76)	0.37 (0.06, 2.23)	0.54 (0.09, 3.02)	0.61 (0.10, 3.49)	0.78 (0.13, 4.49)	0.93 (0.17, 5.29)	**Sorafenib**	*Rank10*									
0.23 (0.02, 2.63)	0.25 (0.02, 3.11)	0.28 (0.02, 3.35)	0.34 (0.03, 4.22)	0.49 (0.04, 5.91)	0.56 (0.05, 6.69)	0.71 (0.06, 8.28)	0.85 (0.07, 10.33)	0.92 (0.16, 5.37)	**Toripabevaci**	*Rank11*								
0.21 (0.02, 2.36)	0.22 (0.02, 2.75)	0.25 (0.02, 2.96)	0.31 (0.03, 3.73)	0.45 (0.04, 5.22)	0.51 (0.04, 6.08)	0.65 (0.05, 7.63)	0.77 (0.07, 9.23)	0.83 (0.14, 4.76)	0.90 (0.08, 11.08)	**Anlopenpuli**	*Rank12*							
0.20 (0.02, 2.47)	0.22 (0.02, 2.85)	0.24 (0.02, 3.03)	0.30 (0.02, 3.87)	0.44 (0.04, 5.33)	0.49 (0.04, 6.19)	0.64 (0.05, 7.97)	0.76 (0.06, 9.23)	0.81 (0.13, 4.98)	0.89 (0.07, 10.96)	0.98 (0.08, 12.22)	**SBRTSora**	*Rank13*						
0.19 (0.02, 2.25)	0.20 (0.02, 2.60)	0.23 (0.02, 2.84)	0.28 (0.02, 3.43)	0.40 (0.03, 4.94)	0.46 (0.04, 5.74)	0.59 (0.05, 7.31)	0.70 (0.06, 8.58)	0.76 (0.13, 4.47)	0.82 (0.07, 9.85)	0.91 (0.08, 10.94)	0.93 (0.08, 11.48)	**SintiliIBI305**	*Rank14*					
0.17 (0.01, 2.10)	0.19 (0.02, 2.37)	0.21 (0.02, 2.52)	0.26 (0.02, 3.23)	0.37 (0.03, 4.36)	0.42 (0.03, 4.88)	0.54 (0.04, 6.39)	0.65 (0.05, 7.92)	0.69 (0.12, 4.05)	0.75 (0.06, 9.38)	0.84 (0.07, 9.93)	0.86 (0.07, 10.81)	0.92 (0.08, 10.72)	**Cabozantinib**	*Rank15*				
0.16 (0.01, 1.98)	0.18 (0.01, 2.28)	0.20 (0.02, 2.42)	0.25 (0.02, 3.00)	0.35 (0.03, 4.26)	0.40 (0.03, 4.86)	0.51 (0.04, 6.22)	0.61 (0.05, 7.56)	0.66 (0.11, 3.89)	0.71 (0.06, 8.95)	0.79 (0.07, 9.76)	0.81 (0.06, 10.31)	0.87 (0.07, 10.59)	0.95 (0.08, 11.07)	**Pexa_vecSora**	*Rank16*			
0.16 (0.01, 1.90)	0.17 (0.01, 2.15)	0.19 (0.02, 2.40)	0.24 (0.02, 2.94)	0.34 (0.03, 4.03)	0.39 (0.03, 4.77)	0.50 (0.04, 6.02)	0.59 (0.05, 7.04)	0.64 (0.11, 3.76)	0.69 (0.06, 8.46)	0.76 (0.06, 9.36)	0.78 (0.06, 9.97)	0.84 (0.07, 10.68)	0.91 (0.08, 11.22)	0.97 (0.08, 11.93)	**SoraHAIC**	*Rank17*		
0.13 (0.01, 1.64)	0.15 (0.01, 1.84)	0.16 (0.01, 2.06)	0.20 (0.02, 2.53)	0.29 (0.02, 3.50)	0.33 (0.03, 3.98)	0.43 (0.03, 5.03)	0.51 (0.04, 6.11)	0.54 (0.09, 3.23)	0.59 (0.05, 7.42)	0.66 (0.05, 7.98)	0.67 (0.05, 8.56)	0.72 (0.06, 8.76)	0.78 (0.06, 9.47)	0.83 (0.07, 9.95)	0.86 (0.07, 10.53)	**FinotSCT510**	*Rank18*	
0.11 (0.01, 1.36)	0.12 (0.01, 1.58)	0.14 (0.01, 1.69)	0.17 (0.01, 2.13)	0.25 (0.02, 2.86)	0.28 (0.02, 3.32)	0.36 (0.03, 4.44)	0.43 (0.04, 5.43)	0.46 (0.08, 2.71)	0.50 (0.04, 6.35)	0.56 (0.05, 6.62)	0.57 (0.05, 7.26)	0.61 (0.05, 7.23)	0.67 (0.11, 3.90)	0.71 (0.06, 8.51)	0.73 (0.06, 8.88)	0.85 (0.07, 10.44)	**Caboatezo**	*Rank19*
0.06 (0.01, 0.78)	0.07 (0.01, 0.87)	0.08 (0.01, 0.94)	0.10 (0.01, 1.16)	0.14 (0.01, 1.62)	0.16 (0.01, 1.86)	0.20 (0.02, 2.41)	0.24 (0.02, 2.89)	0.26 (0.04, 1.51)	0.28 (0.02, 3.37)	0.31 (0.03, 3.58)	0.32 (0.02, 4.07)	0.34 (0.03, 4.07)	0.37 (0.03, 4.47)	0.39 (0.03, 4.64)	0.41 (0.03, 4.87)	0.47 (0.04, 5.87)	0.56 (0.04, 6.97)	**Camrelirivoc**

OR below 1.00 reflects a lower level 3 and above adverse events. Soraerloti, Sorafenib + erlotinib; SoraHAIC, Sorafenib + hepatic arterial infusion chemotherapy; SintiliIBI305, Sintilimab + Bevacizumab biosimilar; STRIDE: Single Tremelimumab Regular Interval Durvalumab; HAIC_FO, Hepatic arterial infusion chemotherapy of infusional fluorouracil; Camrelirivoc, Camrelizumab + rivoceranib; Pexa_vecSora, Pexastimogene devacirepvec + sorafenib; Caboatezo, Cabozantinib + atezolizumab; SBRTSora, Stereotactic body radiation therapy + sorafenib; FinotSCT510, Finotonlimab + bevacizumab biosimilar; Toripabevaci, Toripalimab + bevacizumab; Anlopenpuli, Anlotinib + penpulimab. RE, radioembolization.

Bold font indicates treatment names.

### Cumulative ranking probabilities

**Figure 3:**
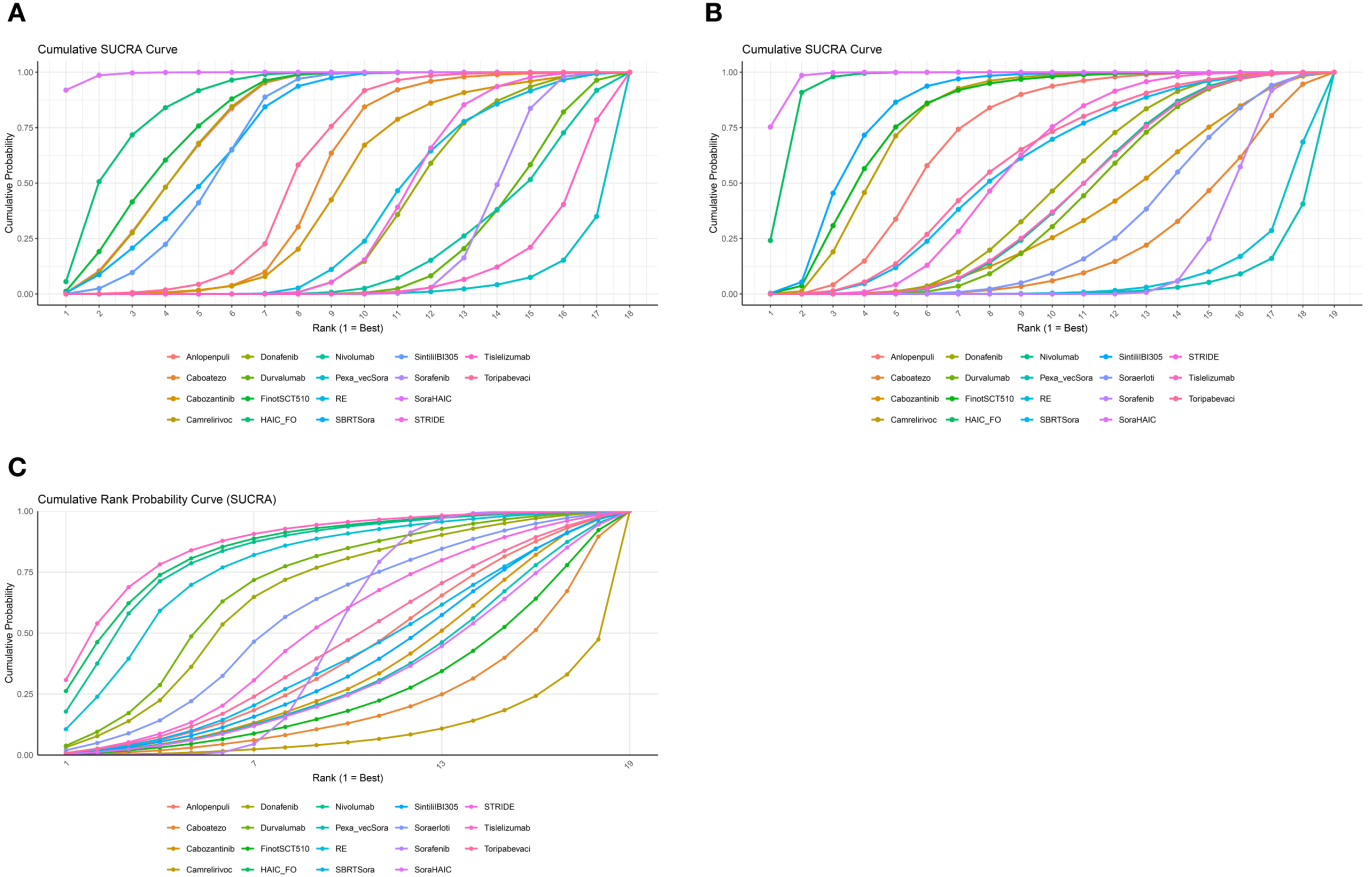
The surface under the cumulative ranking curve summarizes the ranking probabilities of first-line treatments within the network, with higher values indicating a greater likelihood of superior performance. Panels **(A–C)** display treatment rankings for progression-free survival, overall survival, and grade ≥ 3 adverse events, respectively. Soraerloti, Sorafenib + erlotinib; SoraHAIC, Sorafenib + hepatic arterial infusion chemotherapy; SintiliIBI305, Sintilimab + Bevacizumab biosimilar; STRIDE, Single Tremelimumab Regular Interval Durvalumab; HAIC_FO, Hepatic arterial infusion chemotherapy of infusional fluorouracil; Camrelirivoc, Camrelizumab + rivoceranib; Pexa_vecSora, Pexastimogene devacirepvec + sorafenib; Caboatezo, Cabozantinib + atezolizumab; SBRTSora, Stereotactic body radiation therapy + sorafenib; FinotSCT510, Finotonlimab + bevacizumab biosimilar; Toripabevaci, Toripalimab + bevacizumab; Anlopenpuli, Anlotinib + penpulimab; RE, radioembolization.

Based on SUCRA estimates, SoraHAIC and HAIC_FO consistently ranked highest for both progression-free survival and overall survival, with SUCRA values of 99.42% and 87.17% for PFS, and 98.54% and 95.13% for OS, respectively. Other combination strategies demonstrated intermediate rankings, showing heterogeneity across efficacy and safety outcomes. The SUCRA for PFS, OS, and grade ≥ 3 adverse events are shown in **Figures 3A–C**.

### Subgroup analyses

**Figure 4:**
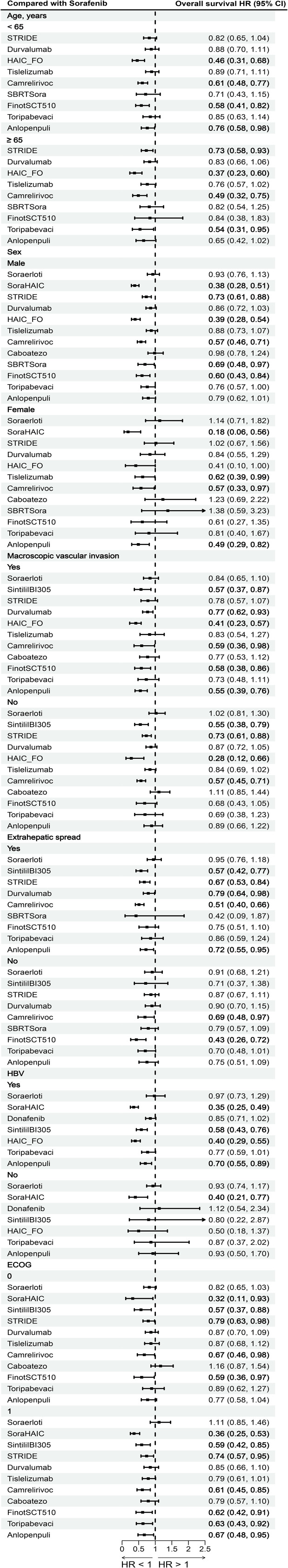
Subgroup analysis of overall survival. Anlopenpuli, Anlotinib + penpulimab; Caboatezo, Cabozantinib + atezolizumab; Camrelizumab + rivoceranib; FinotSCT510, Finotonlimab + bevacizumab biosimilar; HAIC_FO, Hepatic arterial infusion chemotherapy of infusional fluorouracil; SBRTSora, Stereotactic body radiation therapy + sorafenib; SintiliIBI305, Sintilimab + Bevacizumab biosimilar; Soraerloti, Sorafenib + erlotinib; SoraHAIC, Sorafenib + hepatic arterial infusion chemotherapy; STRIDE, Single Tremelimumab Regular Interval Durvalumab; Toripabevaci, Toripalimab + bevacizumab.

Treatment effects were generally consistent across predefined subgroups stratified by age, sex, extrahepatic spread (EHS), macrovascular invasion (MVI), HBV infection, and ECOG performance status, with several immune-based combinations and locoregional approaches showing overall survival (OS) advantages over sorafenib.In patients younger than 65 years, significant OS benefits were observed with HAIC_FO (HR = 0.46, 95% CI: 0.31–0.68), Camrelirivoc (HR = 0.61, 95% CI: 0.48–0.77), FinotSCT510 (HR = 0.58, 95% CI: 0.41–0.82), and Anlopenpuli (HR = 0.76, 95% CI: 0.58–0.98). Among patients aged ≥65 years, survival benefits remained evident with STRIDE (HR = 0.73, 95% CI: 0.58–0.93), HAIC_FO (HR = 0.37, 95% CI: 0.23–0.60), Camrelirivoc (HR = 0.49, 95% CI: 0.32–0.75), and Toripabevaci (HR = 0.54, 95% CI: 0.31–0.95).Sex-specific analyses showed OS improvements in both males and females. In male patients, SoraHAIC (HR = 0.38, 95% CI: 0.28–0.51), HAIC_FO (HR = 0.39, 95% CI: 0.28–0.54), Camrelirivoc (HR = 0.57, 95% CI: 0.46–0.71), STRIDE (HR = 0.73, 95% CI: 0.61–0.88), SBRTSora (HR = 0.69, 95% CI: 0.48–0.97), and FinotSCT510 (HR = 0.60, 95% CI: 0.43–0.84) were associated with improved OS. In female patients, SoraHAIC (HR = 0.18, 95% CI: 0.06–0.56), Tislelizumab (HR = 0.62, 95% CI: 0.39–0.99), Camrelirivoc (HR = 0.57, 95% CI: 0.33–0.97), and Anlopenpuli (HR = 0.49, 95% CI: 0.29–0.82) demonstrated significant survival advantages. Across MVI-defined subgroups, HAIC_FO, Camrelirivoc, and SintiliIBI305 consistently reduced mortality risk regardless of vascular invasion status. Similar consistency was observed across EHS subgroups, with Camrelirivoc showing survival benefits both in patients with (HR = 0.51, 95% CI: 0.40–0.66) and without extrahepatic spread (HR = 0.69, 95% CI: 0.48–0.97).In HBV-positive patients, SoraHAIC (HR = 0.35, 95% CI: 0.25–0.49), HAIC_FO (HR = 0.40, 95% CI: 0.29–0.55), SintiliIBI305 (HR = 0.58, 95% CI: 0.43–0.76), and Anlopenpuli (HR = 0.70, 95% CI: 0.55–0.89) showed significant OS improvements, whereas among HBV-negative patients, only SoraHAIC retained a clear survival advantage (HR = 0.40, 95% CI: 0.21–0.77). Similar to the findings across HBV-defined subgroups, SoraHAIC maintained a consistent survival benefit in both ECOG 0 and ECOG 1 populations (**Figure 4**).

## Discussion

This Bayesian network meta-analysis provides a comparative evaluation of first-line treatments for advanced or unresectable locally advanced hepatocellular carcinoma. Across randomized evidence, several novel regimens demonstrated clear survival advantages over sorafenib, with HAIC-based strategies—particularly sorafenib plus HAIC and HAIC_FO—achieving the most favorable rankings for both progression-free and overall survival while maintaining acceptable safety profiles.

Beyond HAIC-based approaches, multiple immunotherapy-containing combinations showed clinically meaningful benefits. Regimens combining immune checkpoint inhibitors with anti-angiogenic agents or tyrosine kinase inhibitors improved survival outcomes to varying degrees, although their performance differed across efficacy and toxicity endpoints. Notably, STRIDE improved overall survival without a corresponding gain in progression-free survival, underscoring the heterogeneity in benefit–risk profiles across treatment strategies.

Although the overall incidence of grade ≥3 adverse events was generally acceptable, immune-related adverse events (irAEs) remain clinically important in patients receiving immune checkpoint inhibitor-based therapies. Previous studies have shown that irAEs may affect multiple organ systems and, in some cases, occur after prolonged treatment exposure or even following treatment discontinuation (36, 37). Therefore, early recognition, regular monitoring, and timely multidisciplinary management are essential to optimize treatment safety and maintain therapeutic benefit, particularly in patients with underlying chronic liver disease or cirrhosis. Nevertheless, currently available long-term safety data remain limited, precluding comprehensive assessment of delayed immune-related toxicities in the present study. Future studies with longer follow-up and more comprehensive safety reporting are warranted to further improve the clinical management of immune-based therapies in advanced HCC.

Subgroup analyses further refined these findings. HAIC_FO and camrelizumab plus rivoceranib consistently conferred survival benefits across age categories and macrovascular invasion status. SoraHAIC and camrelizumab-based combinations demonstrated robust efficacy irrespective of sex, while camrelizumab plus rivoceranib retained benefit regardless of extrahepatic spread. Survival improvements were also observed across ECOG performance status strata, supporting the applicability of selected regimens in patients with preserved functional status.

From a biological perspective, the efficacy of HAIC-based strategies may relate to enhanced intratumoral drug delivery and immune modulation. Accumulating evidence suggests that combining locoregional therapies with sorafenib produces synergistic antitumor effects in hepatocellular carcinoma (38). Consistently, our analysis demonstrates that HAIC plus sorafenib yields marked improvements in both progression-free and overall survival among patients with advanced or unresectable locally advanced disease. Mechanistically, HAIC may exert immunomodulatory effects beyond cytotoxic drug delivery. Tertiary lymphoid structures (TLSs), particularly intratumoral TLSs, are increasingly recognized as favorable prognostic markers and facilitators of antitumor immunity, whereas peritumoral TLSs may be functionally impaired (39). Notably, HAIC has been shown to induce intratumoral TLS formation via CXCL12-dependent mechanisms, thereby reshaping the tumor immune microenvironment toward an antitumor state (40).

Immune escape and aberrant angiogenesis represent hallmark features of hepatocellular carcinoma and critically influence treatment outcomes (41). Tumor-mediated immune suppression through regulatory T-cell recruitment and immune checkpoint upregulation provides a strong biological rationale for combining immune checkpoint inhibitors with anti-angiogenic agents (42, 43). In line with this concept, our results indicate that immunotherapy plus anti-angiogenic therapy significantly prolongs PFS and OS compared with sorafenib monotherapy, with consistent benefits across ECOG status, macrovascular invasion, and extrahepatic spread subgroups. Sorafenib pioneered systemic therapy for unresectable HCC through multi-target kinase inhibition, but the therapeutic landscape has since shifted with the advent of immune checkpoint inhibitors, newer TKIs, and rational combination strategies (44, 45). Our findings further support the superiority of immunotherapy–TKI combinations over sorafenib alone across both the overall cohort and multiple clinical subgroups, without a corresponding increase in severe toxicity.

Microsphere-based embolization is a treatment approach that achieves antitumor effects by selectively blocking the tumor-feeding vasculature (46). By contrast, microsphere-based embolization combined with sorafenib did not confer significant survival advantages in this analysis, possibly reflecting heterogeneity in microsphere characteristics and patient selection. Similarly, immune monotherapy showed no statistically significant survival benefit over sorafenib, although modest OS trends were observed, underscoring the importance of appropriate patient stratification. Therefore, these findings also should be interpreted with caution in the context of other clinical factors.

Given the strong etiological role of chronic HBV infection in HCC (47), its predominance in younger Asian populations (48), and the adverse prognostic impact of macrovascular invasion (49), subgroup analyses were performed to address clinical heterogeneity. These analyses suggest that anti-angiogenic therapy combined with immunotherapy is particularly beneficial in patients with HBV infection, macrovascular invasion, female sex, and age younger than 65 years.

Although viral hepatitis has long been the predominant etiological factor for HCC worldwide, the increasing burden of metabolic-related diseases has led to a marked rise in the prevalence of nonalcoholic fatty liver disease and its progressive form, nonalcoholic steatohepatitis, which are increasingly recognized as major contributors to HCC in developed countries (50). Distinct etiological backgrounds may not only shape the molecular characteristics and tumor immune microenvironment of HCC, but also influence treatment responsiveness and subsequent clinical outcomes (51, 52). However, owing to the limited subgroup data available from the included trials, we were unable to perform a network meta-analysis stratified comprehensively by geographic region and etiology. Therefore, the external generalizability of the present findings to populations not predominantly affected by HBV-related HCC should be interpreted with caution. We also hope that future studies will incorporate regional and etiological stratification to further improve the generalizability of the findings and more accurately characterize treatment heterogeneity across diverse HCC populations. In addition, inevitable heterogeneity in baseline patient characteristics across the included studies may have influenced the pooled estimates. Accordingly, caution should be exercised when interpreting and applying these results in clinical practice, particularly across different patient populations and clinical settings. Furthermore, all comparator arms included in this study were sorafenib-based, and RCTs using other agents as comparators were not captured. Furthermore, certain phase III randomized trials—such as the CheckMate 9DW study (53) were excluded because survival outcomes for the sorafenib and lenvatinib control subgroups could not be separately extracted. This exclusion may introduce potential bias in estimating the true effectiveness of dual-immunotherapy for patients with advanced or locally advanced HCC. Although the network meta-analysis allows indirect comparisons across multiple treatment strategies, some relevant evidence—particularly RCTs with non-sorafenib control groups—may still have been missed. Therefore, when applying the conclusions of this study to clinical decision-making, it is essential to integrate the most up-to-date clinical research findings to ensure a comprehensive evaluation and to minimize potential bias arising from incomplete evidence.

It is important to note that the development of new drugs and clinical trials in the field of liver cancer is progressing rapidly. Some of the treatments that have been included have received new follow-up data in recent studies or have been updated by other evidence; at the same time, emerging new treatment modalities and combination strategies may further change the current treatment landscape. Therefore, the conclusions drawn in this study based on network Meta-analysis should be comprehensively considered within the evolving evidence system, combined with the latest clinical research and updates in guidelines, to support more precise clinical decisions.

## Conclusion

This network meta-analysis synthesizes evidence from randomized controlled trials to compare first-line systemic treatment strategies for advanced and unresectable locally advanced hepatocellular carcinoma. HAIC-based regimens emerged as the most effective options in terms of progression-free and overall survival, while several immunotherapy-based combinations also achieved clinically relevant survival benefits across heterogeneous patient subgroups without compromising safety. Together, these findings highlight the potential advantages of combining locoregional interventions with systemic and immune-based therapies in contemporary HCC treatment. Nonetheless, as current evidence is constrained by methodological limitations and an evolving therapeutic landscape, future head-to-head randomized trials and updated network meta-analyses are necessary to further define optimal first-line treatment strategies.

## Data Availability

Publicly available datasets were analyzed in this study. This data can be found here: The datasets analyzed in this study were derived from previously published articles cited in the reference list. No single repository or accession number applies to this secondary analysis. The source data are publicly available in the original publications and, where applicable, in the corresponding trial registry records.
