# Supplementary material for: Comparative efficacy and safety of first-line treatments for advanced hepatocellular carcinoma: a Bayesian network meta-analysis
Source: Front Immunol. 2026 Jun 5;17:1846603. doi: 10.3389/fimmu.2026.1846603 (PMC13279430; doi:10.3389/fimmu.2026.1846603)

**Supplementary Figure 1.** Risk-of-bias derived from the ROB2 assessment. Risk-of-bias plot (A), summary plot (B).

A


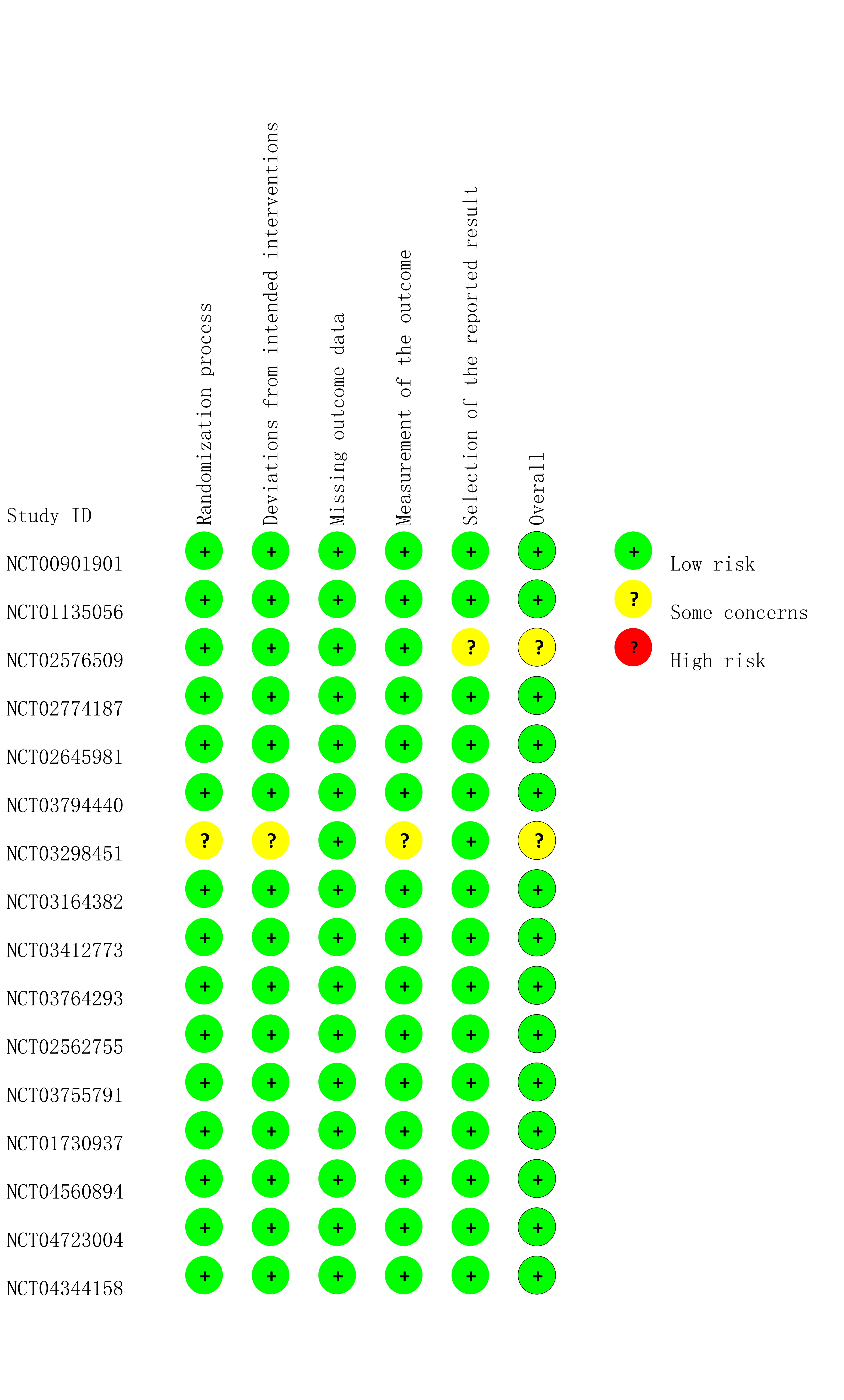


B


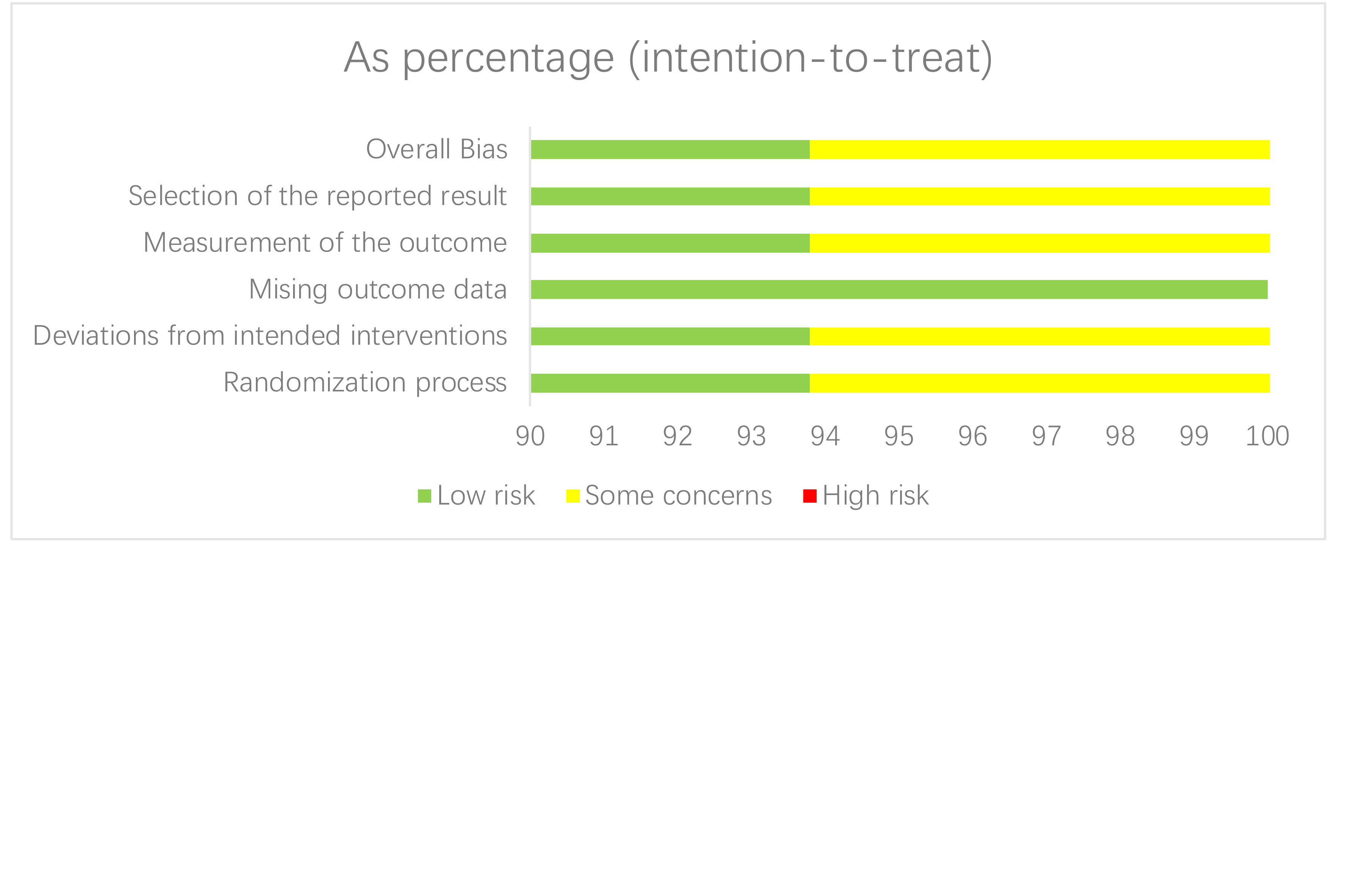


**Supplementary Figure 2.** Trace plots illustrating convergence of the three Markov chain Monte Carlo (MCMC) chains for progression-free survival (A), overall survival (B), and grade ≥ 3 adverse events (C).

A


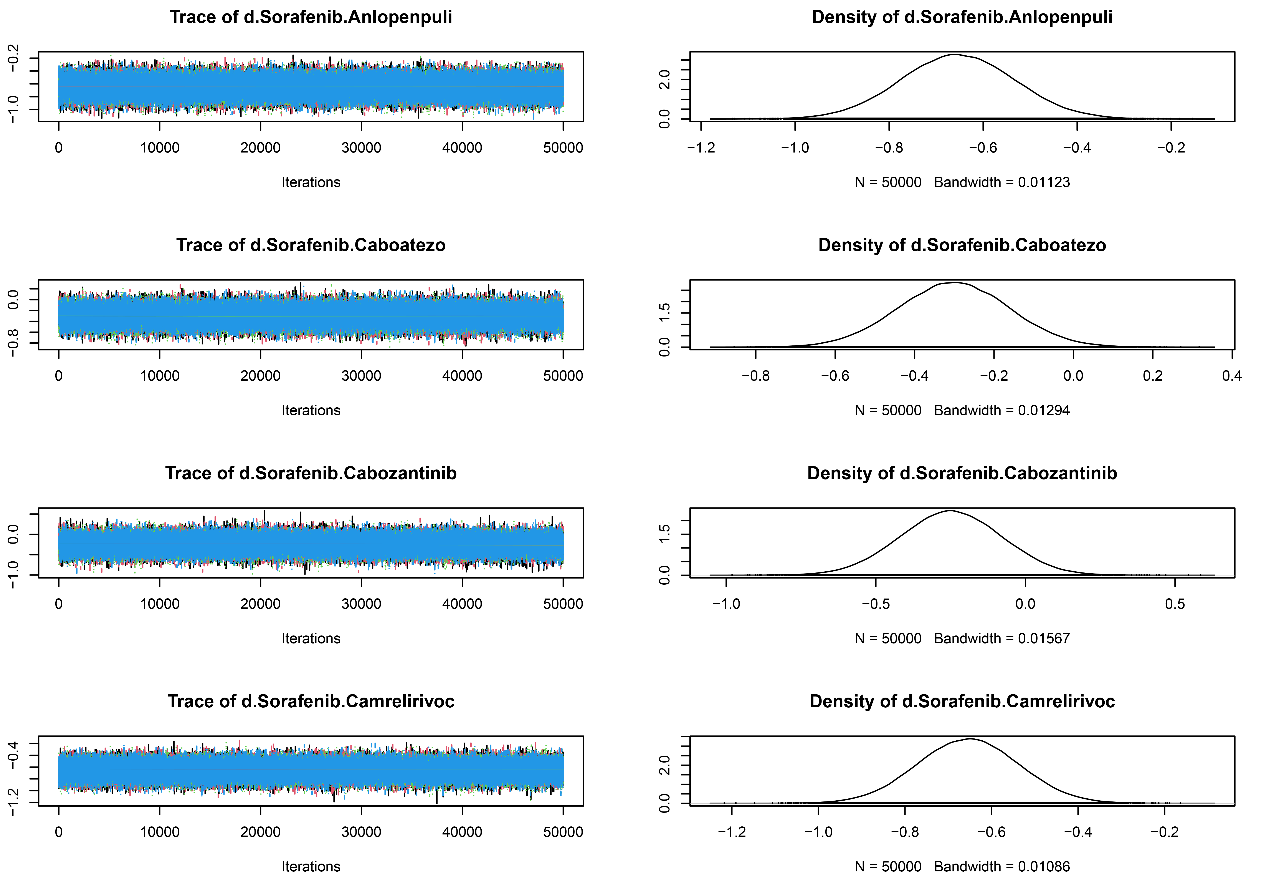


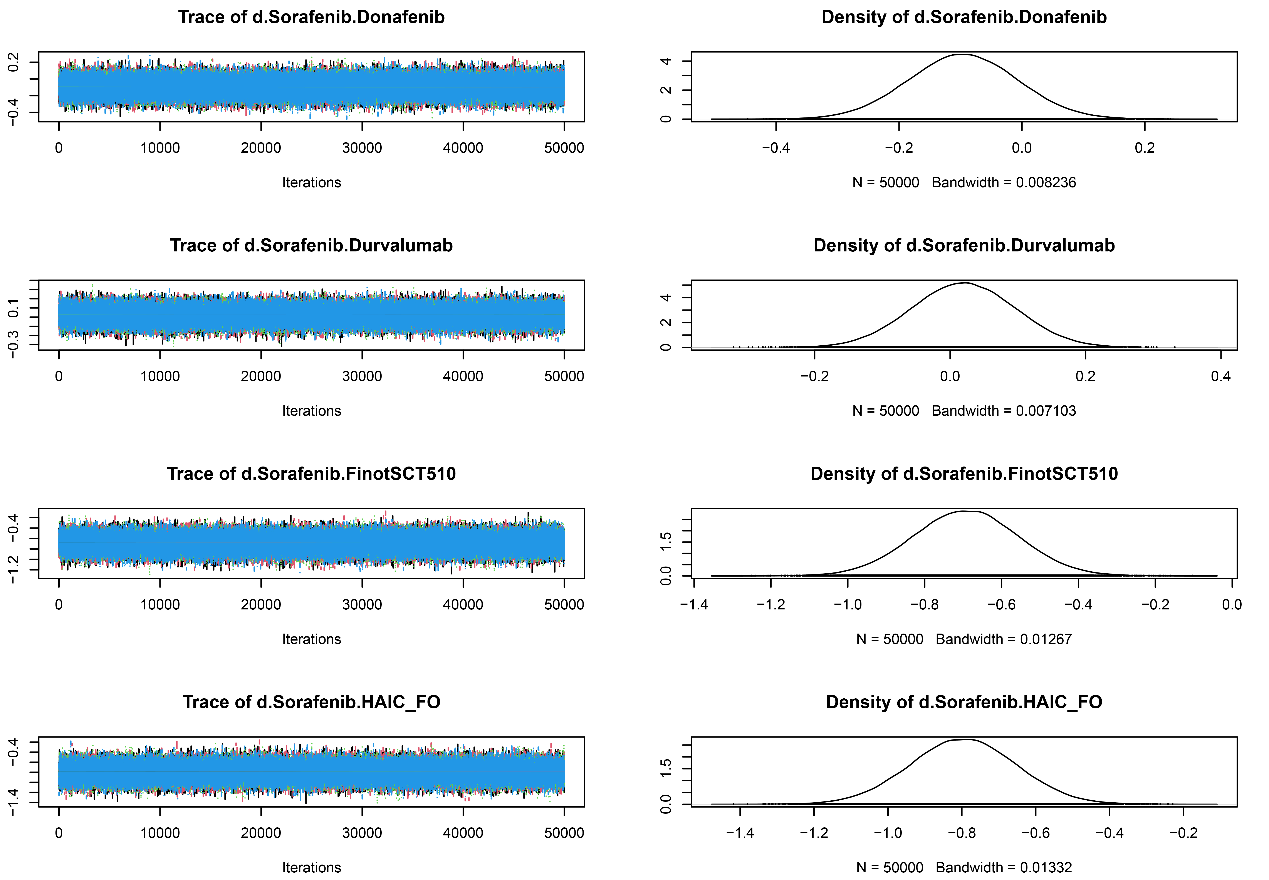

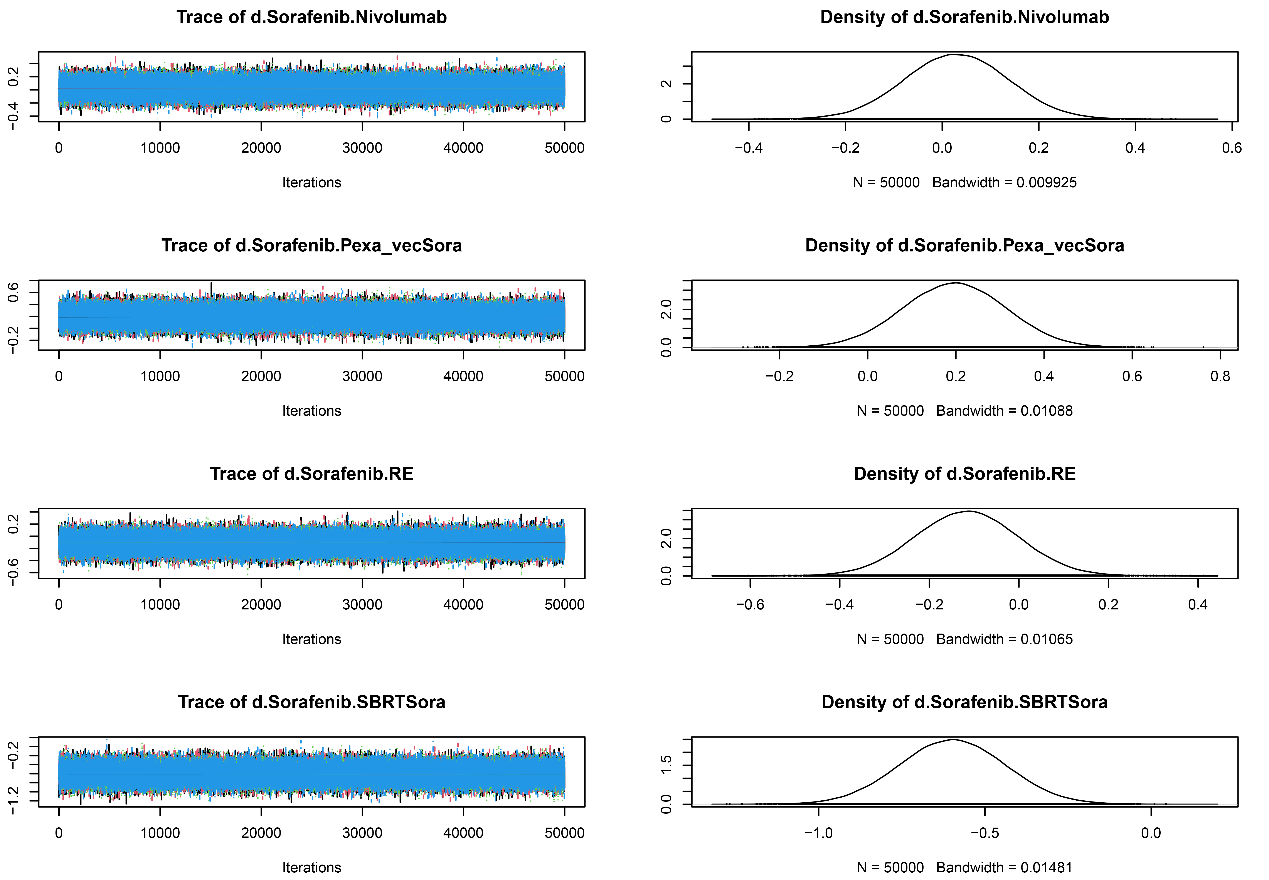

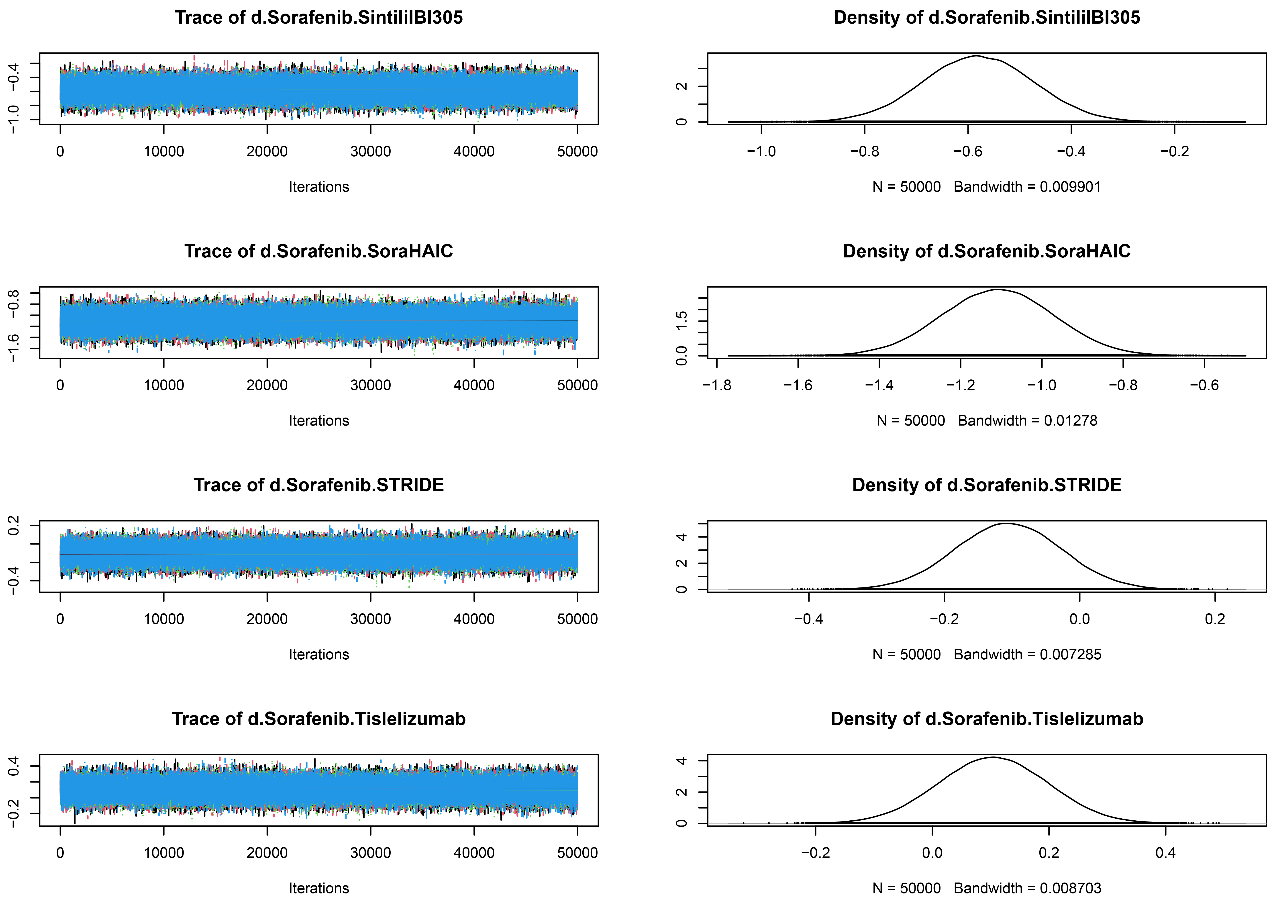

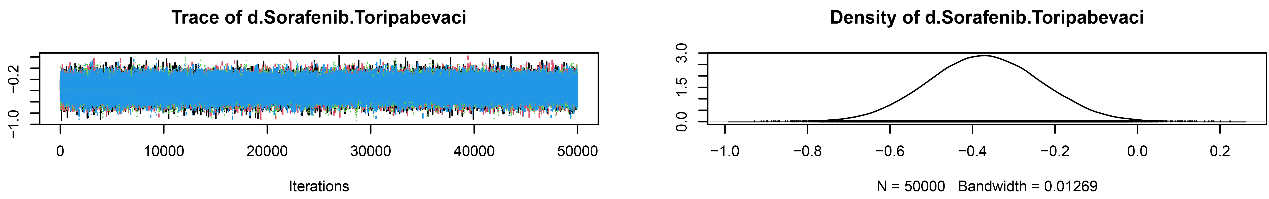


B


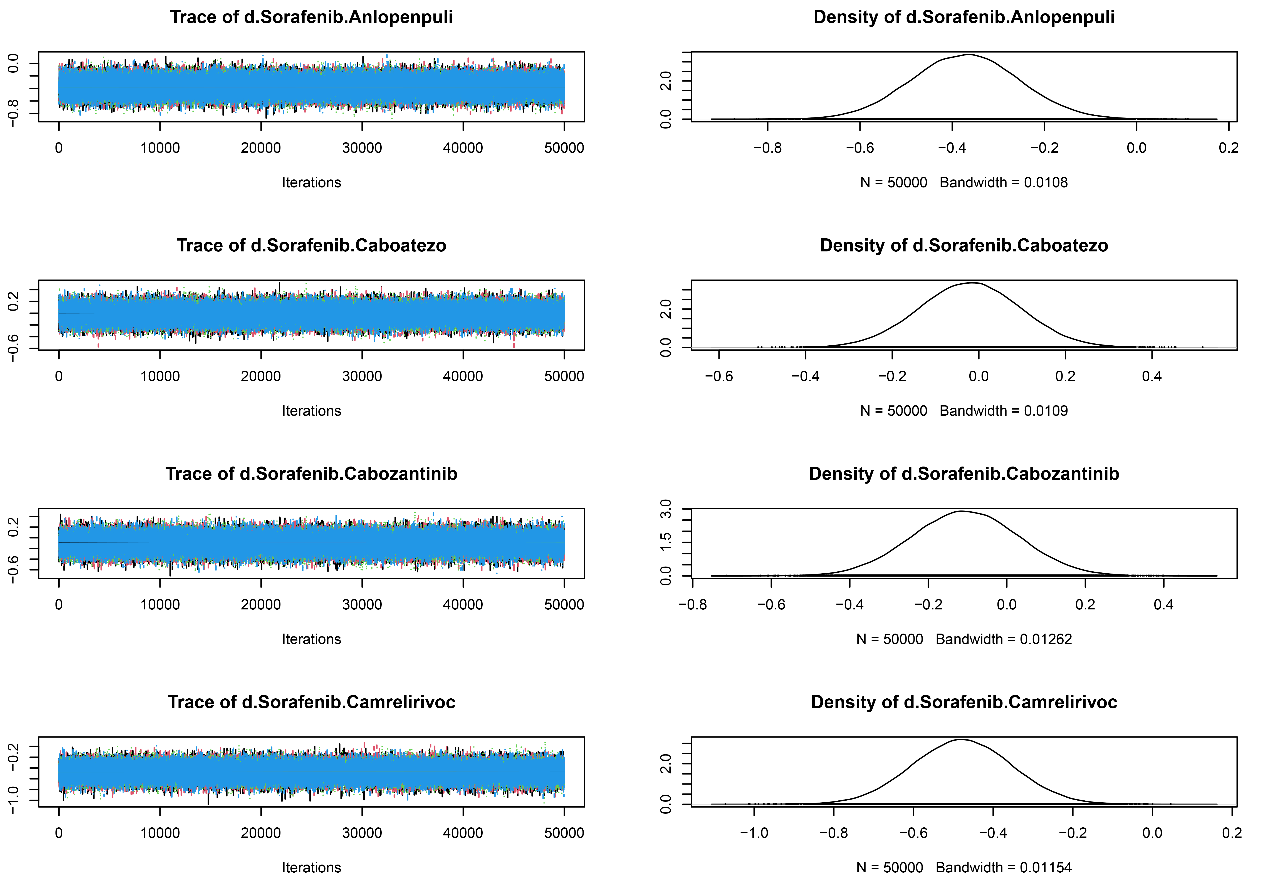

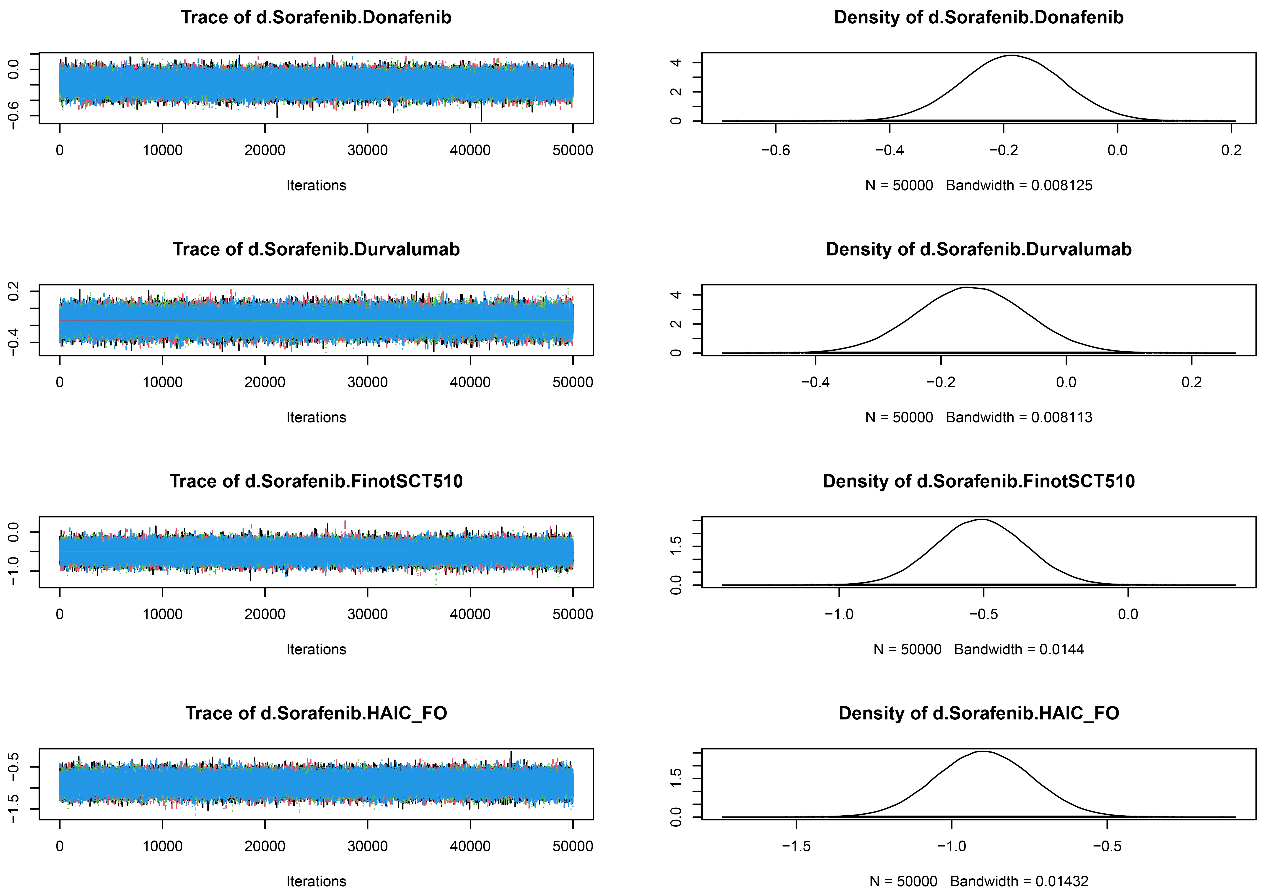

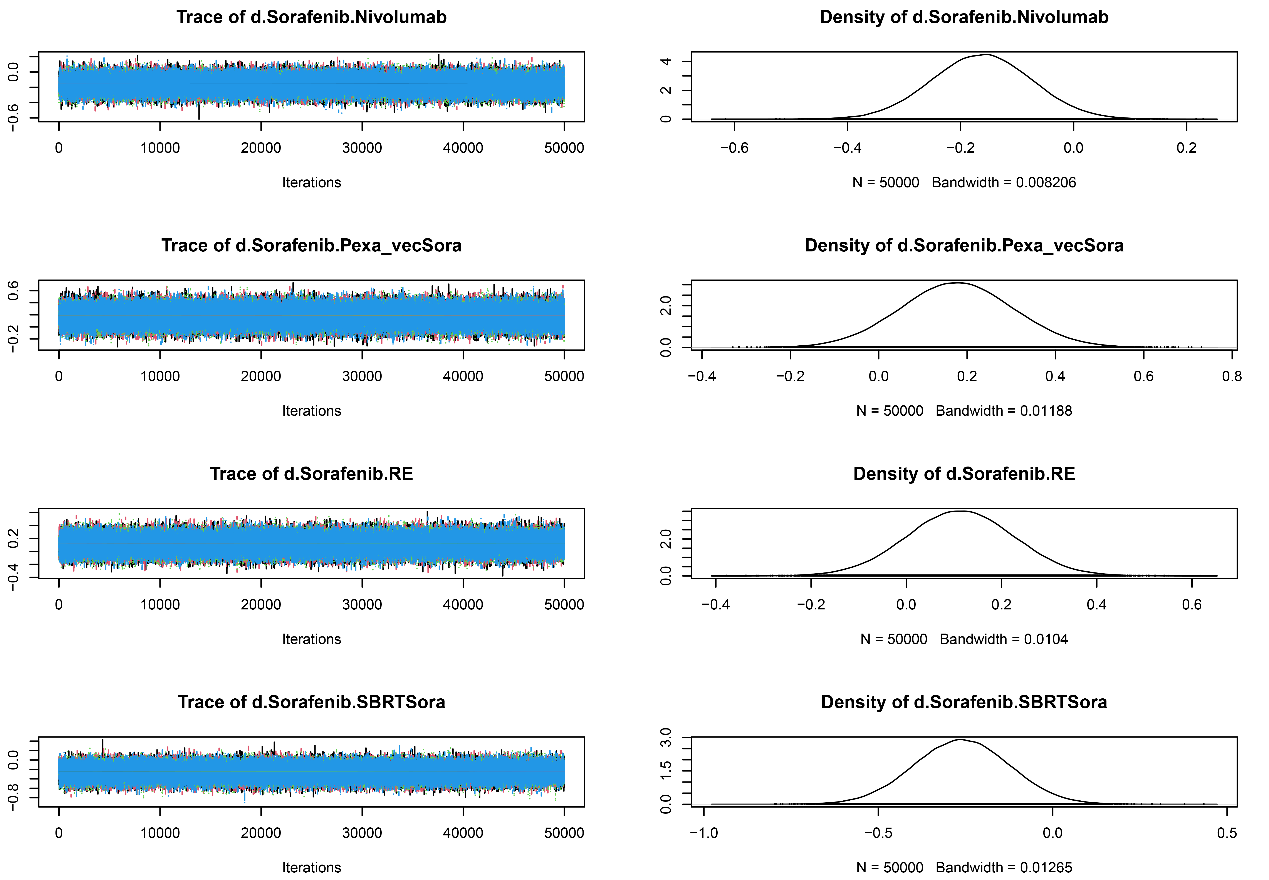

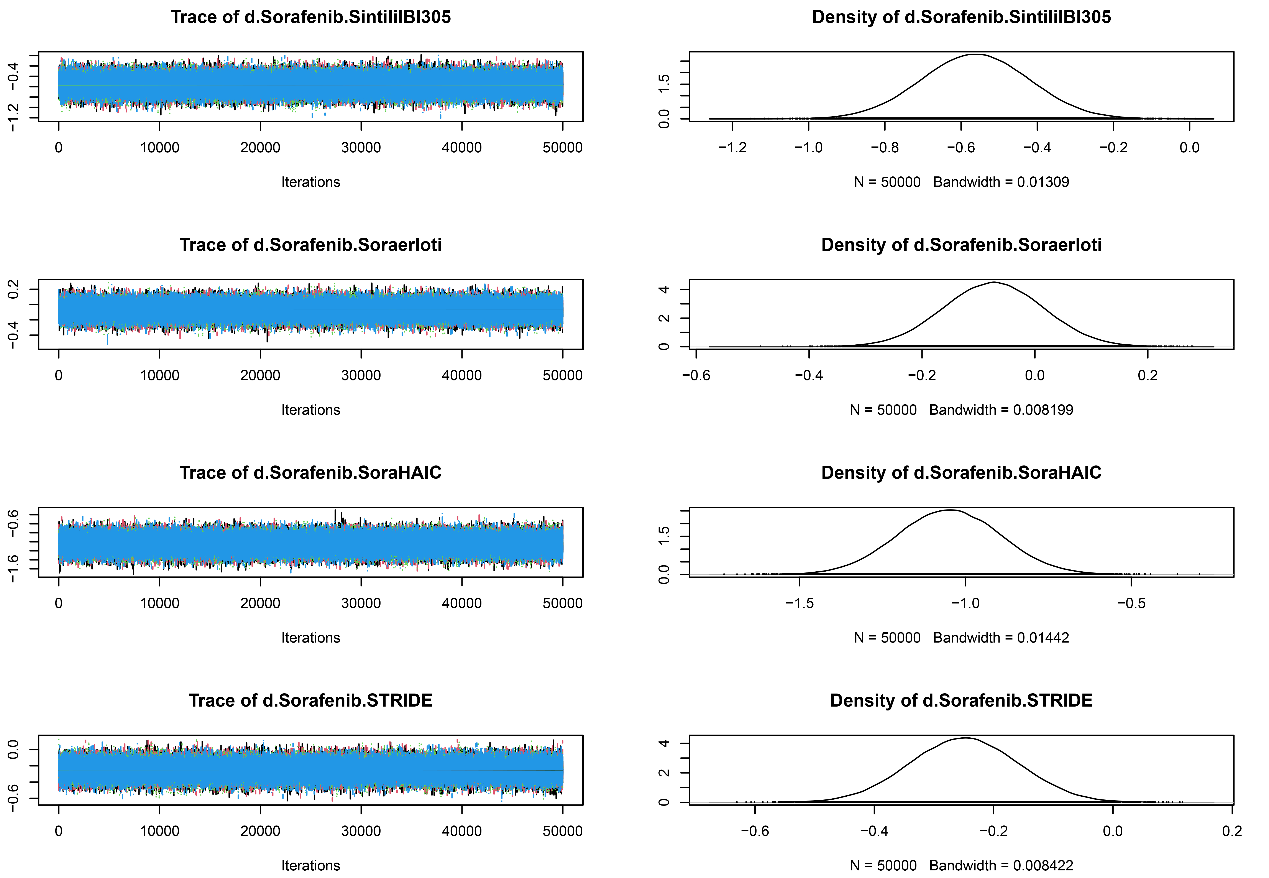

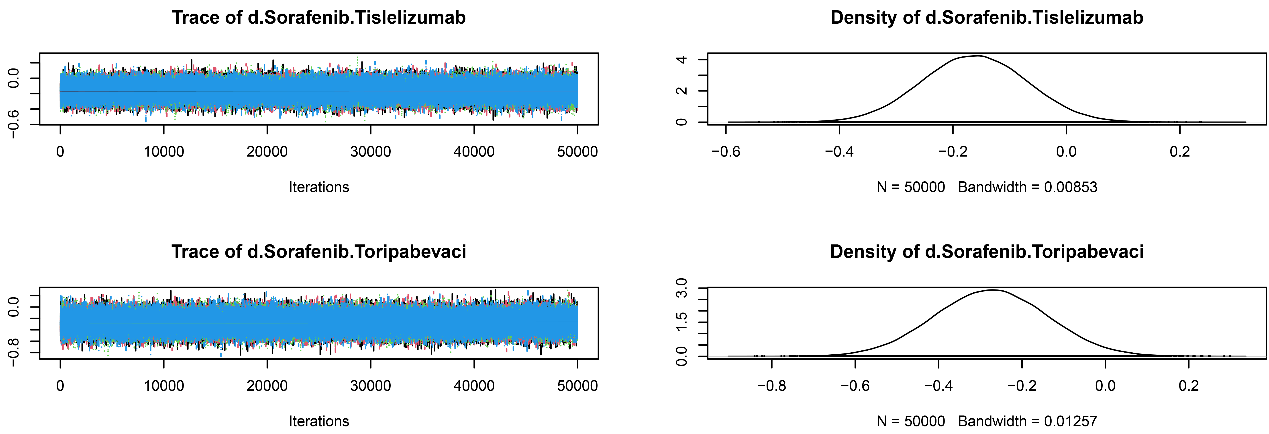


C


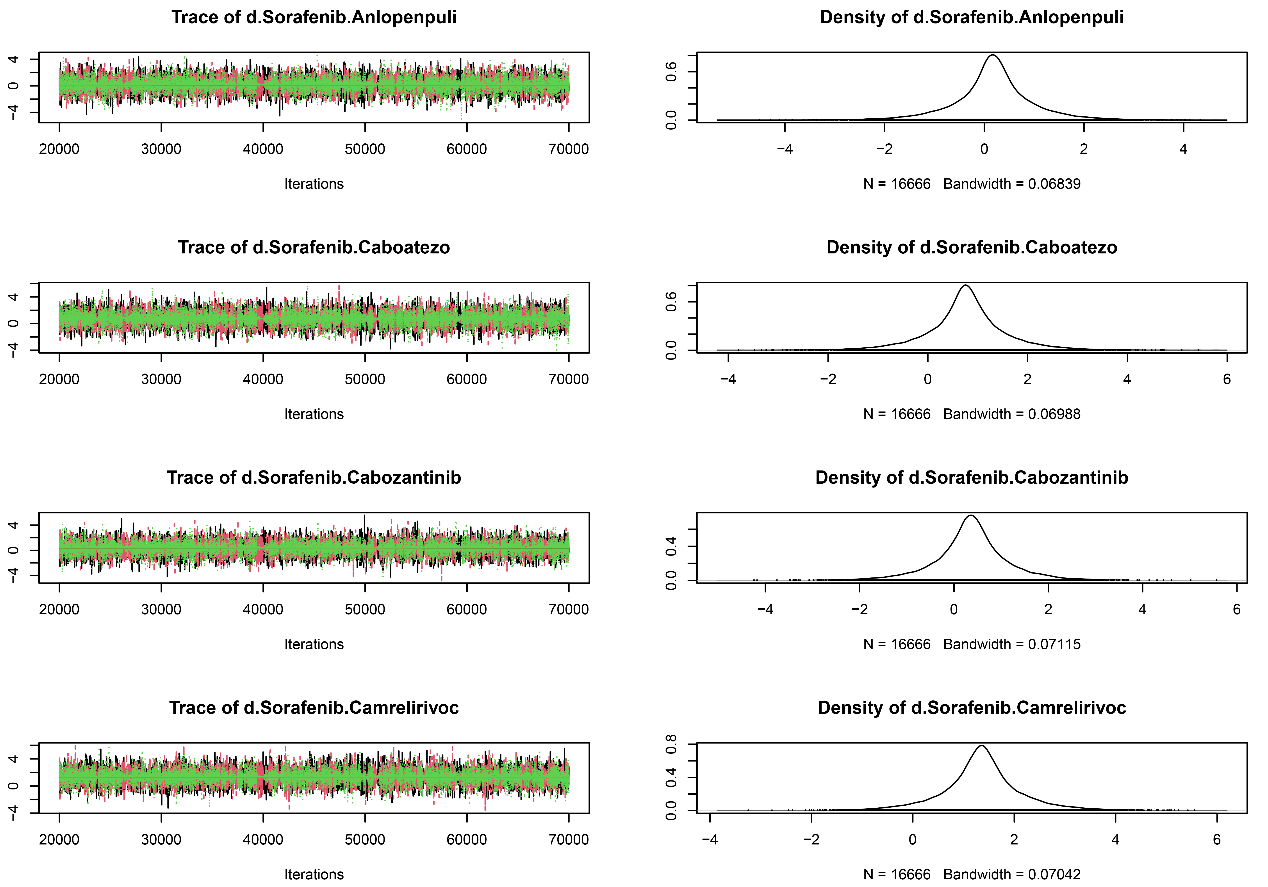

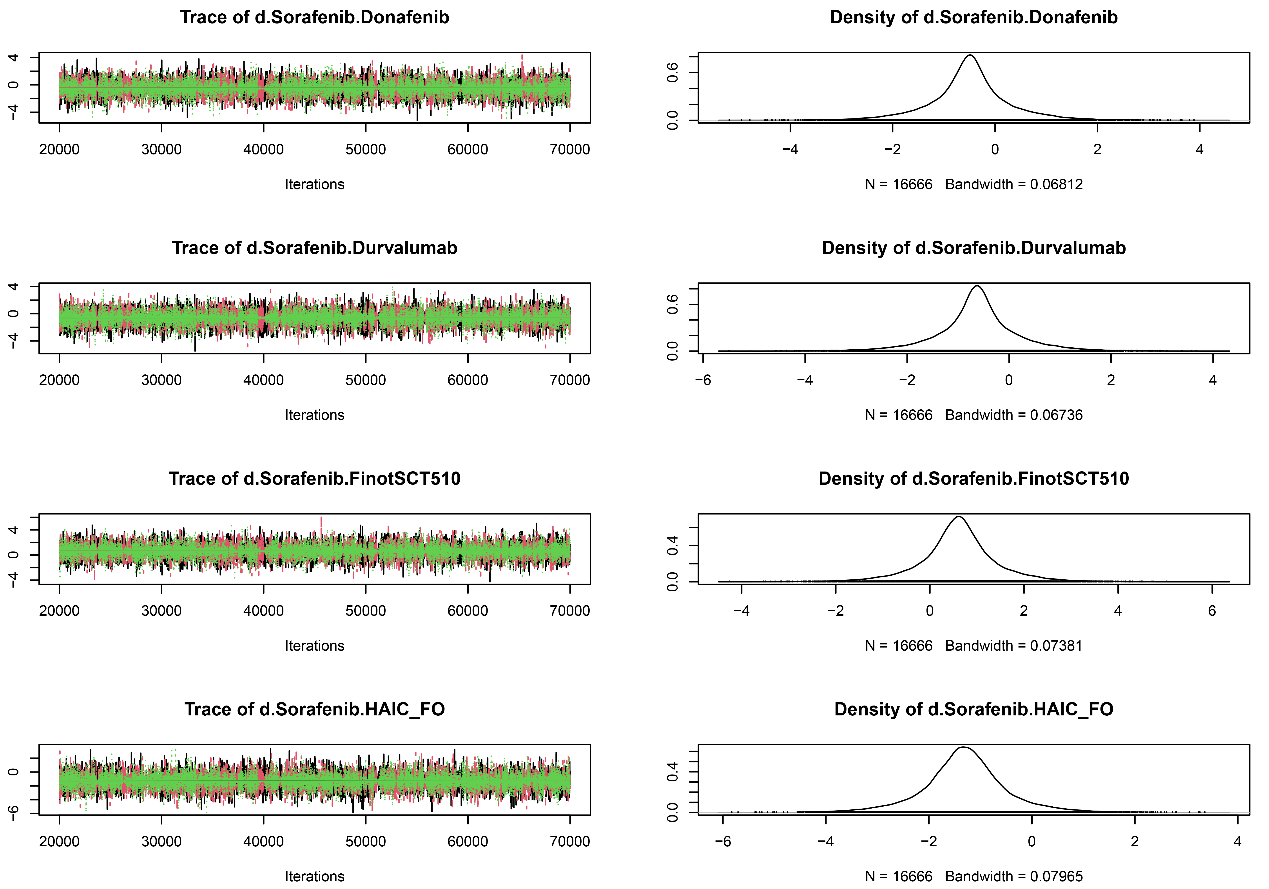

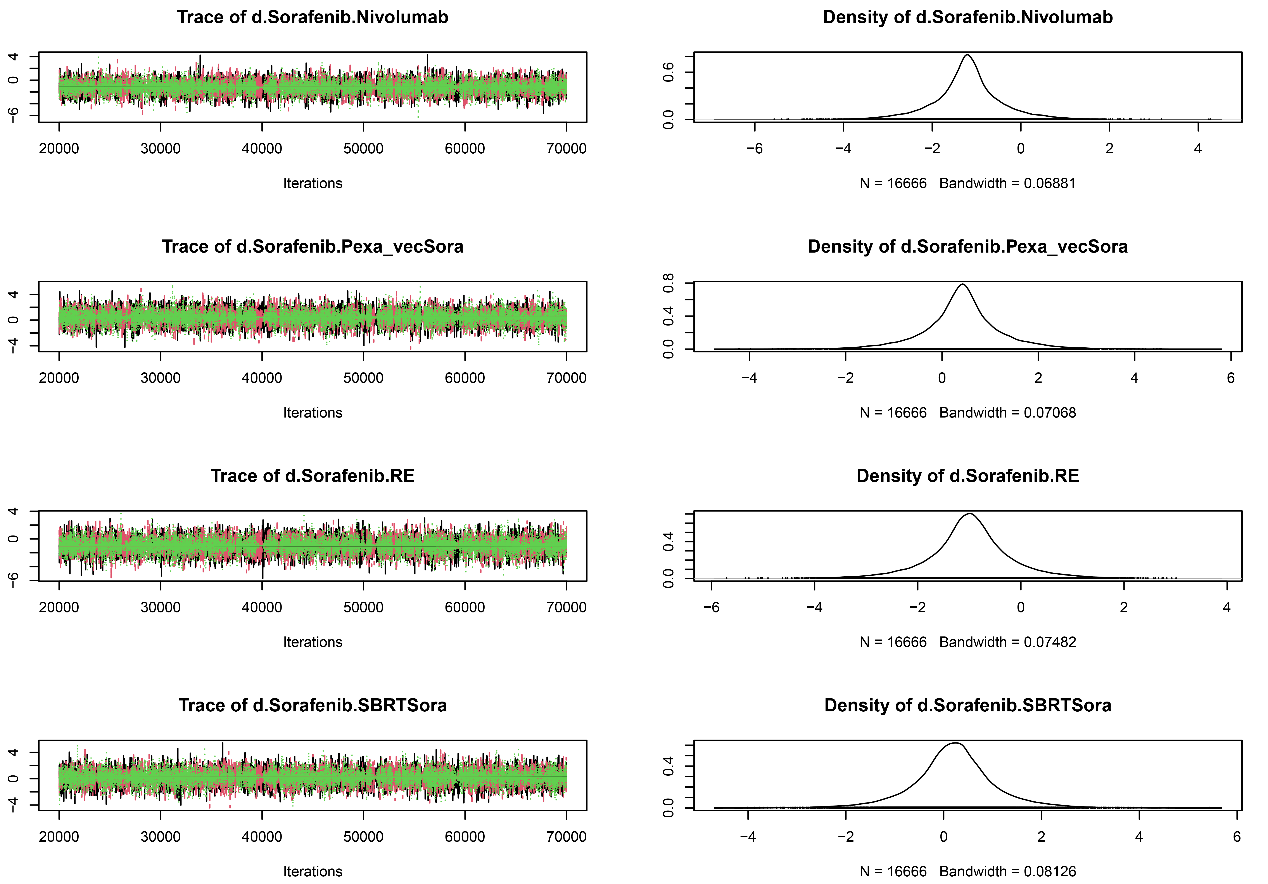

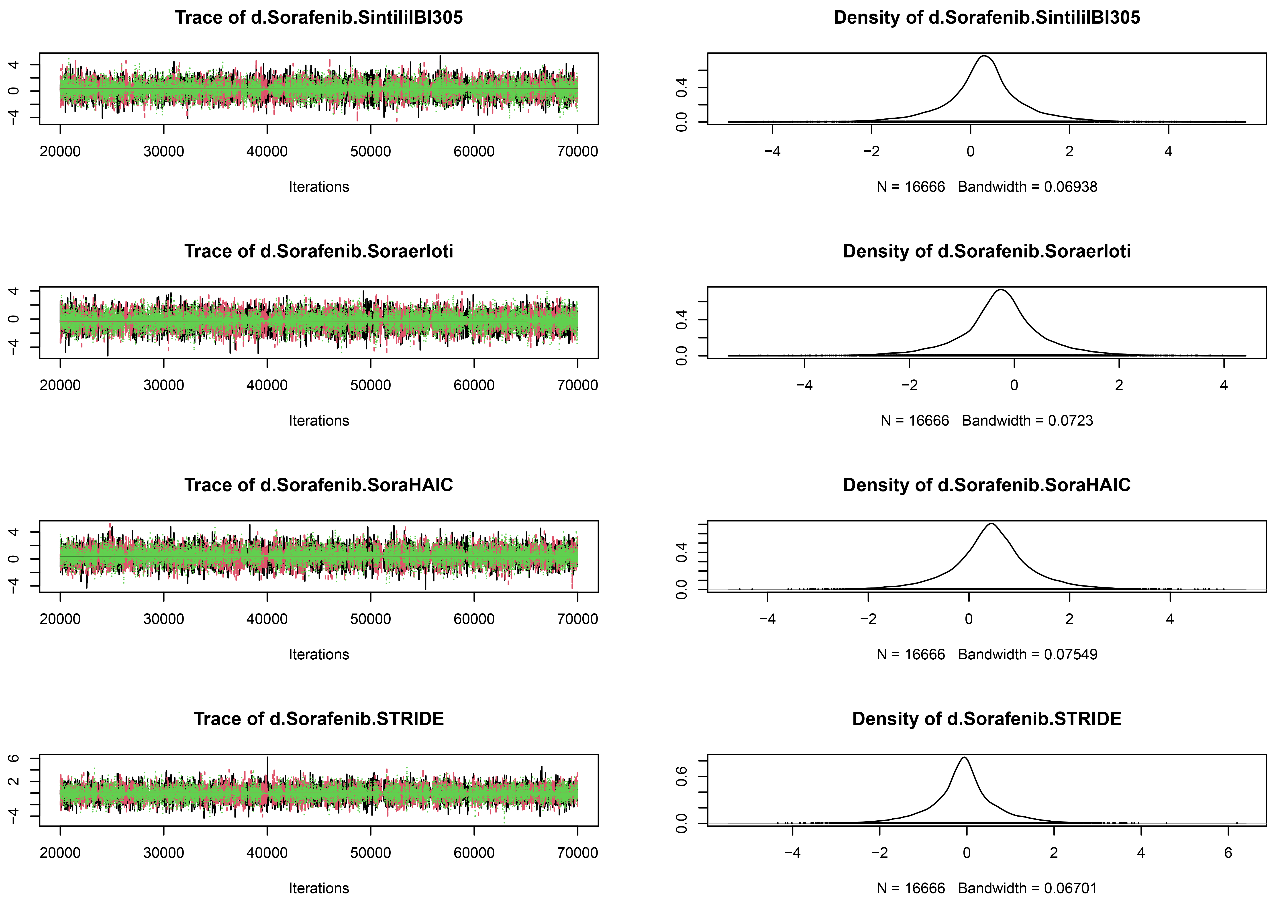

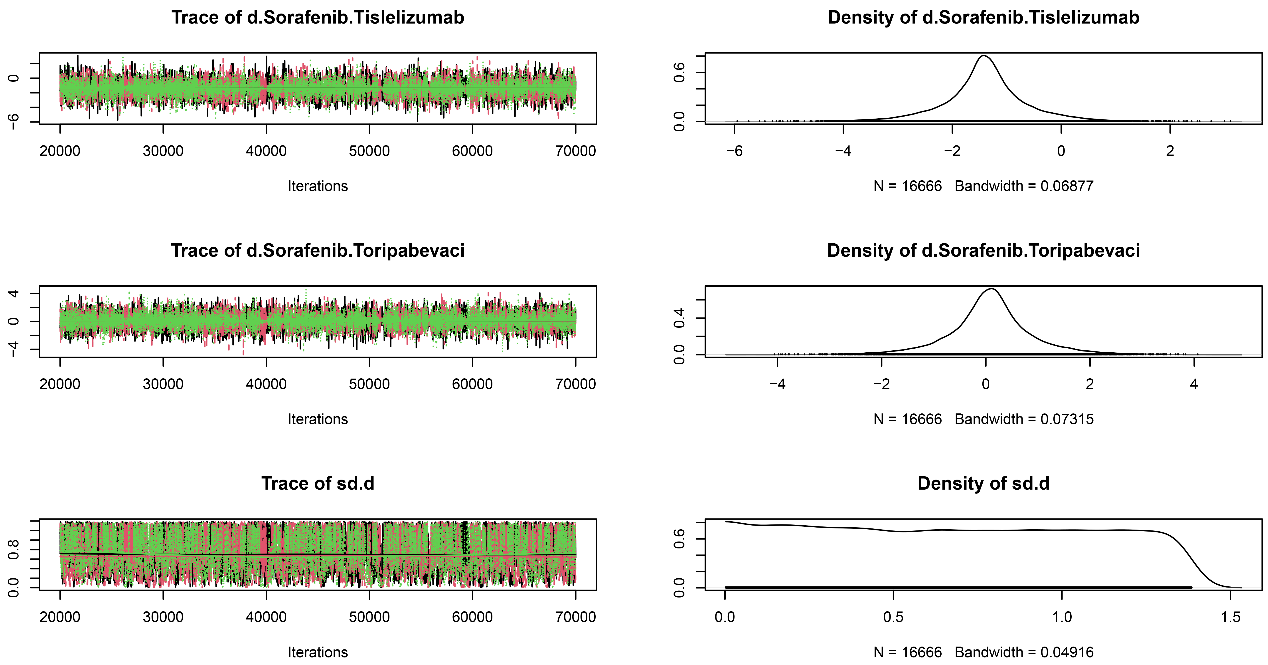


**Supplementary Figure 3.** Assessment of model convergence using the Brooks–Gelman–Rubin diagnostic, showing satisfactory convergence of the three Markov chain Monte Carlo (MCMC) chains for progression-free survival (A), overall survival (B), and grade ≥ 3 adverse events (C).

A


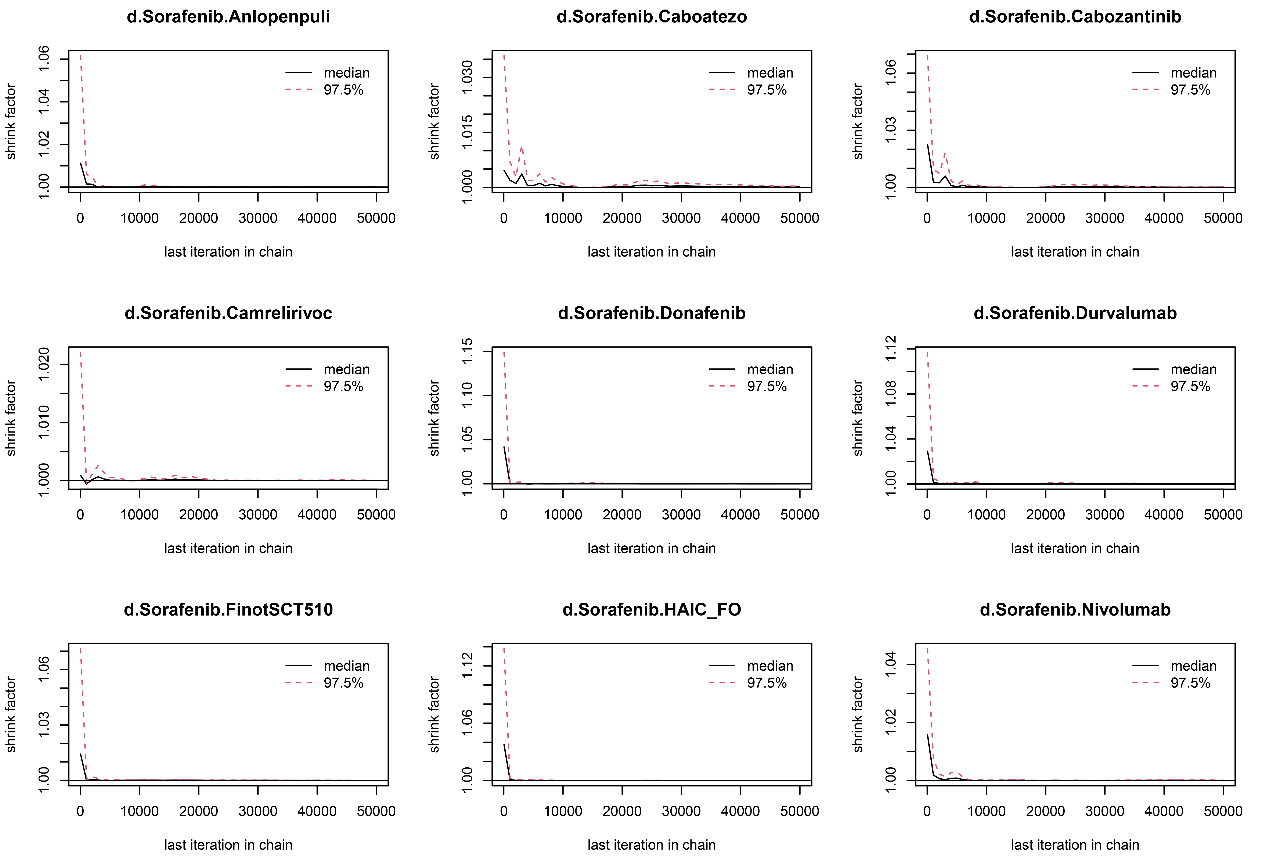

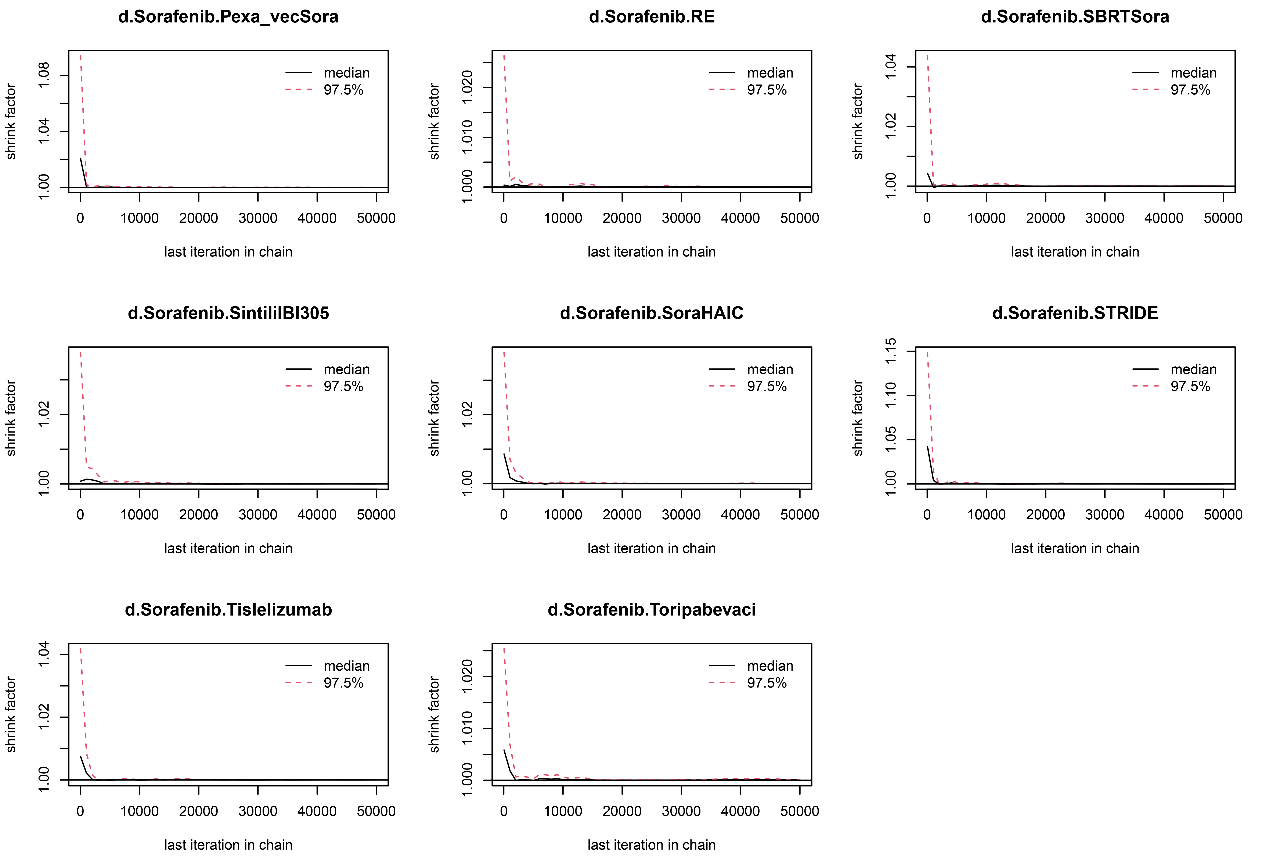


B


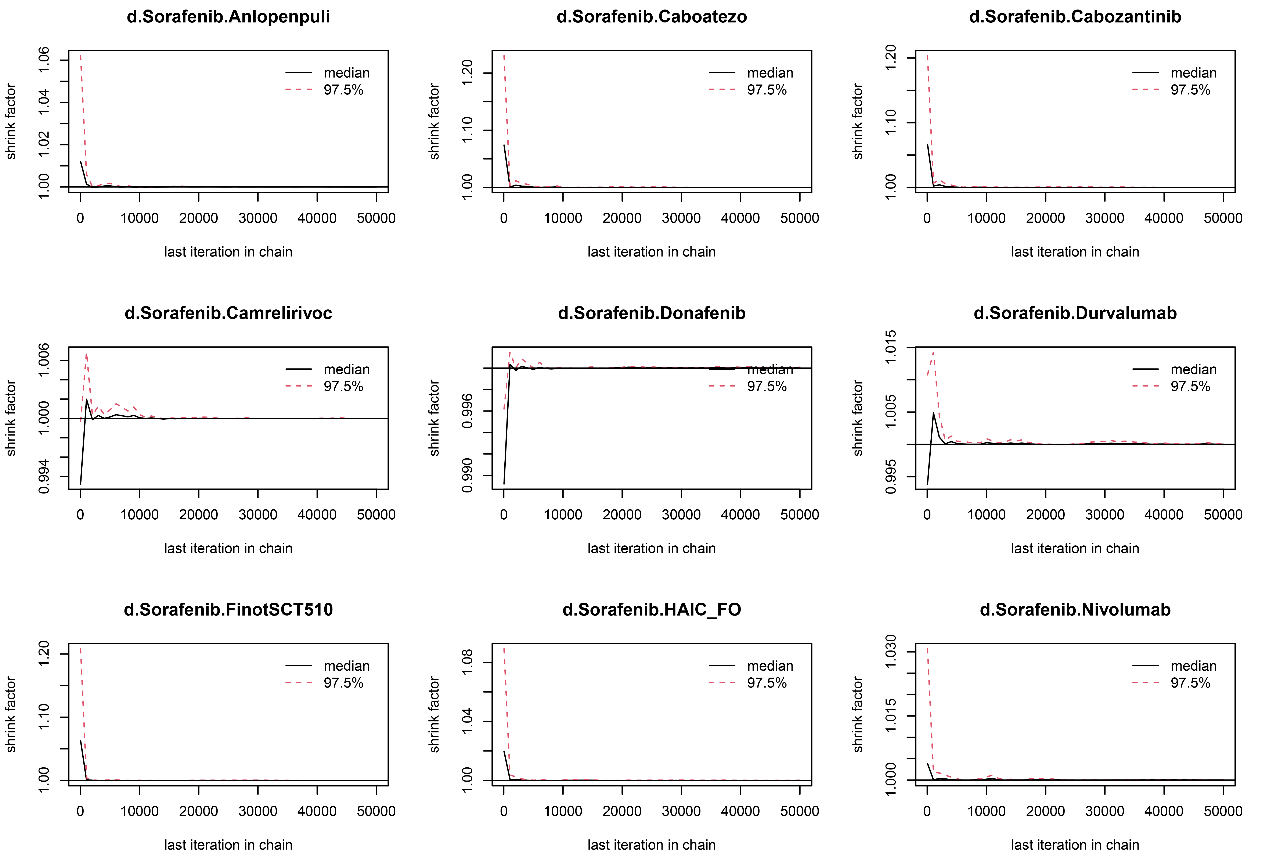


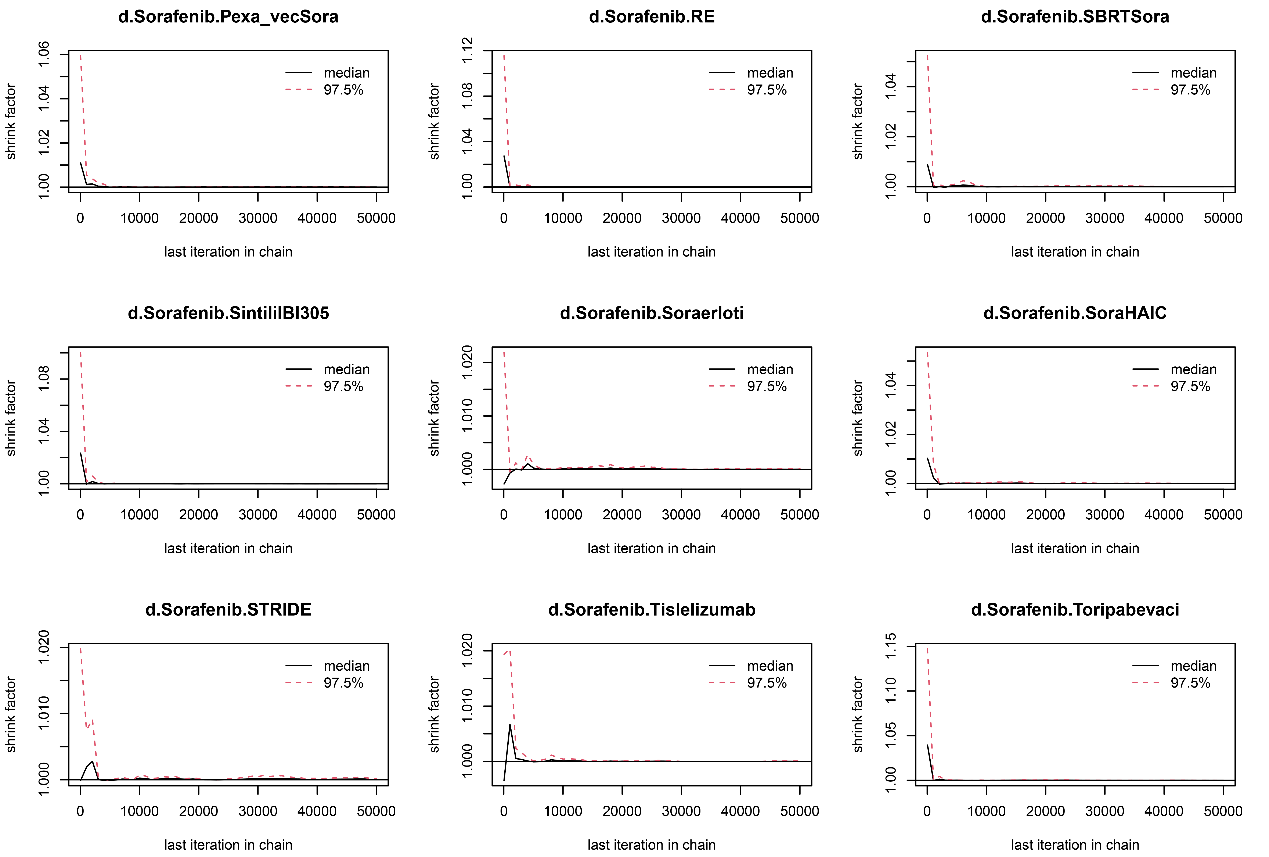


C


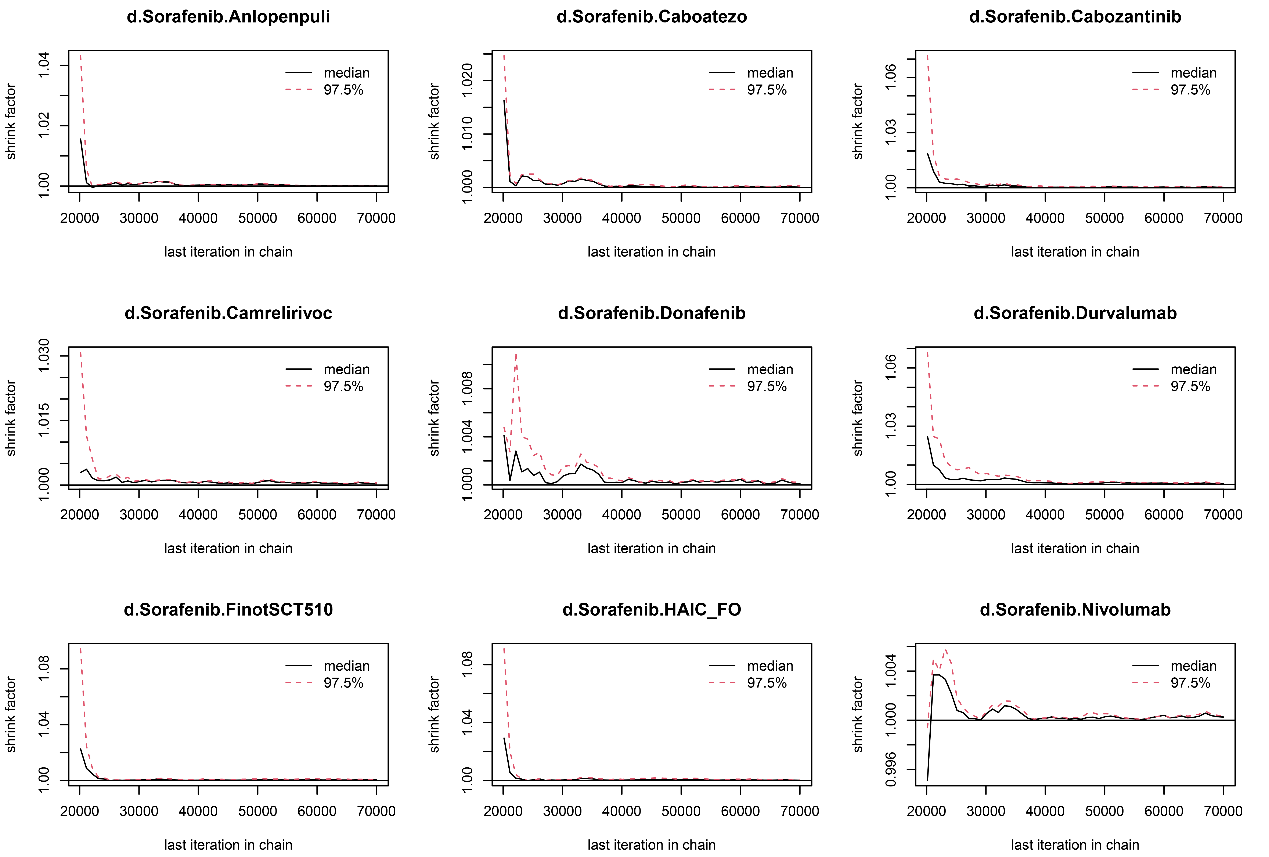


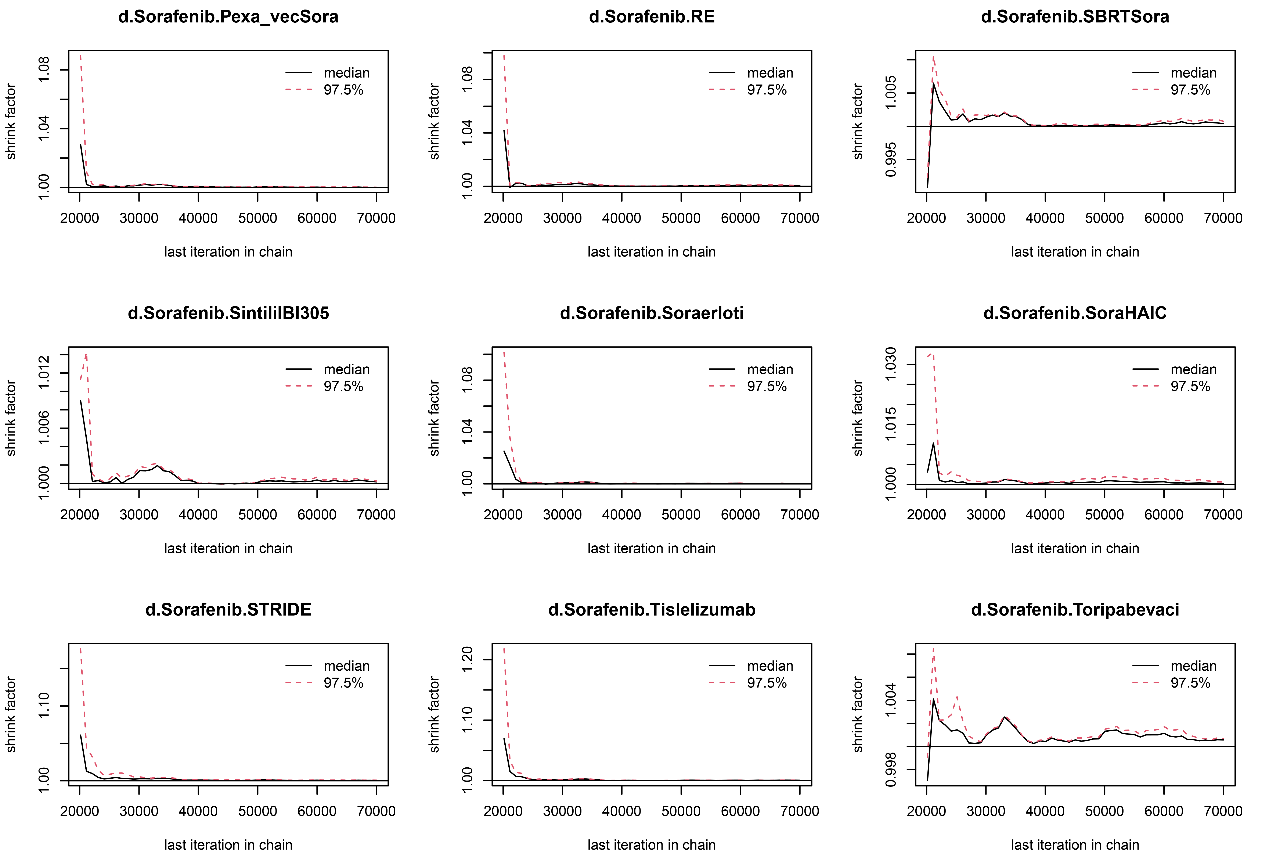

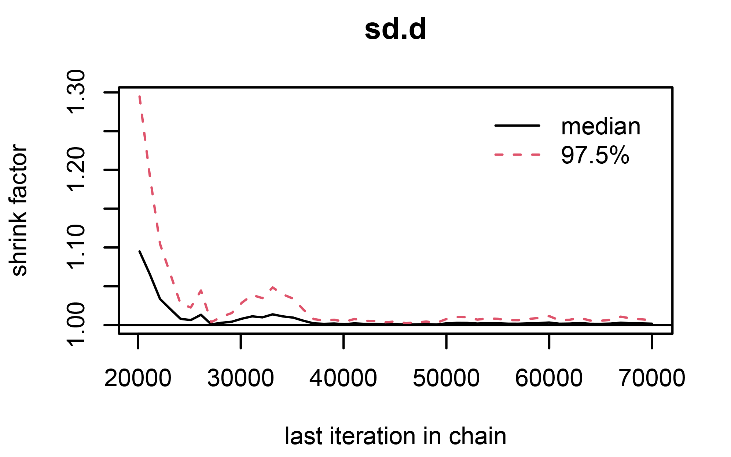

Supplement: Supplementary file 1 [file Table1.docx]
